# Quantifying Individual Health Status from Multi-omics Data by Health State Manifold

**DOI:** 10.1007/s43657-024-00188-4

**Published:** 2025-12-15

**Authors:** Xinyan Zhang, Chengming Zhang, Yanpu Wu, Xu Lin, Xiaoping Liu, Luonan Chen

**Affiliations:** 1 Key Laboratory of Systems Biology, Key Laboratory of Systems Health Science of Zhejiang Province, School of Life Science, Hangzhou Institute for Advanced Study, University of Chinese Academy of Sciences, Hangzhou, 310024 China; 2 Key Laboratory of Systems Biology, Shanghai Institute of Biochemistry and Cell Biology, Center for Excellence in Molecular Cell Science, Chinese Academy of Sciences, Shanghai, 200031 China; 3 School of Life Science and Technology, ShanghaiTech University, Shanghai, 201210 China; 4 West China Biomedical Big Data Center, West China Hospital, Sichuan University, Chengdu, 610041 China

**Keywords:** Personalized health, Homeostatic resilience, Metabolic health, Dynamic network

## Abstract

Quantifying individual health status from increasingly accumulated omics data is essential for both early prevention and intervention of diseases, which attracts great attention from communities of biology and medicine. Most of the existing approaches mainly classify individuals into different catalogues or classes based on phenotypes and biomarkers. However, an individual's health status from a dynamical systems viewpoint can be viewed as a non-equilibrium steady state, which can generally be characterized by two key features, i.e. (1) homeostatic potential that represents the ability of homeostatic resilience to withstand perturbations or maintain functions at the current state/phenotype of this individual and (2) phenotypic potential that represents the state/phenotype of the individual on the whole process from health to disease. Here, we proposed a health state manifold (HSM) method derived from dynamic network biomarker method and diffusion map theory to quantify individual health status with the characterization of such two features in a robust and accurate manner based on multi-omics data. To verify our method, HSM method was applied to the quantification of diabetes mellitus (rat subjects) and the Roux-en-Y Gastric Bypass (human subjects) for both disease progression process and recovery process, which demonstrated its effectiveness and potential for personalized medicine and preventive medicine.

**Supplementary Information:**

The online version contains supplementary material available at 10.1007/s43657-024-00188-4.

## Introduction

Nutrition, lifestyle, environment and genetics as determinants influence health status throughout lifespan of each individual. In recent decades, disease patterns have changed with public health measures such as improved nutrition, hygiene, sanitation and more powerful healthcare interventions. The number of people with chronic diseases, e.g. obesity and diabetes, is increasing year by year in worldwide (Huber et al. 2011; Saeedi et al. 2019). The global diabetes prevalence in 2019 is estimated to be 9.3% (463 million people), rising to 10.2% (578 million) by 2030 and 10.9% (700 million) by 2045 (Saeedi et al. 2019). Between 1975 and 2014, the prevalence of obesity (body-mass index (BMI) ≥ 30 kg/m^2^) increased from 3.2% to 10.8% in adult men and from 6.4% to 14.9% in adult women (Collaboration 2016), which substantially increases the risk of diseases such as type 2 diabetes mellitus (T2DM), fatty liver disease, hypertension, myocardial infarction, stroke, dementia, osteoarthritis, obstructive sleep apnea and several cancers, thereby contributing to a decline in both quality of life and life expectancy (Bluher 2019). Even in the some slums the mortality pattern is burdened by chronic diseases increasingly (Kanungo et al. 2010). Aging with chronic disease has become the norm (Huber et al. 2011), and the cost of chronic diseases accounts for the most of expenditures of healthcare system, which takes huge pressure on the sustainability of healthcare system (Huber et al. 2011). At present, early evaluation or diagnosis is crucial for prevention and intervention of chronic diseases, and many studies have shown that intervention in the early stages of chronic disease can greatly reduce the incidence of disease (Li et al. 2008; Nathan and Diabetes Prevention Program 2015).

Hence, it is of great importance to develop a new method for quantifying individual health status from the measure data, which has the potential to contribute to prevent or intervene in the early stage of diseases. In biomedical research, health is commonly defined in a negative fashion as the absence of disease (Conti 2018). Given the overwhelming multiplicity of disease-inducing conditions and pathways, this definition of health as the nonexistence of any kind of pathology is impractical (Ayres 2020). Another key feature of health is the ability to adapt upon a large variety of perturbations (Huber et al. 2011; van Ommen et al. 2009). Recently, many researchers have focused on some biomarkers that can reflect homeostasis in the body, which are named flexibility or resilience (Liu et al. 2022; Wopereis et al. 2017). And a recent review has reported that homeostatic resilience can be one of the hallmarks of individual health (Lopez-Otin and Kroemer 2021).

According to these features of health, the traditional approach to test individual health is to measure static biomarkers that distinguish disease state from normal state mainly based on the differentially expressed molecules on individuals (Liu et al. 2013). But because healthy individuals and pre-disease individuals (i.e. healthy individuals near the disease state) are similar in terms of molecular expression or phenotype, this approach usually fails to distinguish pre-disease individuals from healthy individuals, and therefore does not work for early prevention and intervention of the disease (Liu et al. 2013). The European Food Safety Authority formally proposed an idea of measuring "homeostasis" to evaluate health in 2017 (Hardy et al. 2017). A body has the ability to maintain relatively stable homeostasis in response to stress, i.e., homeostatic resilience. Homeostatic resilience can be used to describe the stability of an individual in the current state, and it means that individuals with lower homeostatic resilience are more likely to transfer into other states when disturbed. PhenFlex test (PFT), which clearly shows the changes of molecular expression of an individual in response to perturbance, is a common method to measure homeostatic resilience (van den Broek et al. 2017; van Ommen et al. 2009). It has been reported that measuring homeostatic resilience by PFT enabled the discrimination between different states of health (van den Broek et al. 2017). However, there is still a lack of effective computational methods for robustly quantifying individual health status based on increasingly accumulated omics data.

**Fig. 1:**
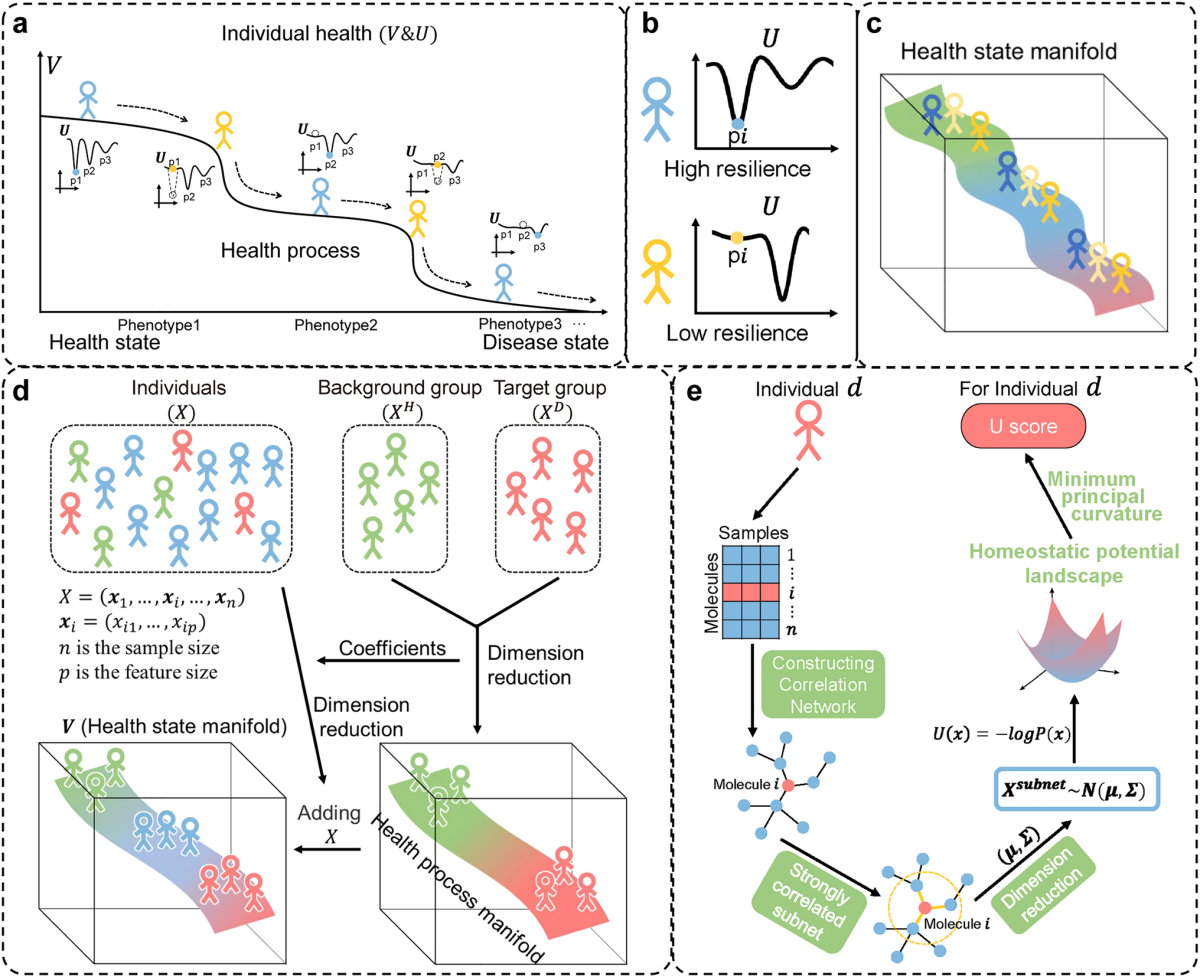
Quantifying individual health status by V and U. **a** Health status of an individual is a multi-stage process from health to disease state with gradual state change in each stage but occasionally a drastic state transition between two stages. An individual's health consists of two parts, one is the individual's current position in the whole health process characterized by V score, and the other is the individual's stability in the current state characterized by U score. **b** Individuals with high homeostatic resilience or potential are stable at current state, and individuals with low homeostatic resilience or potential are prone to transfer into other state. **c** The health difference not only for the individuals even with a similar phenotype by homeostatic potential, but also for the individuals even with a similar homeostatic potential by health state manifold. **d** Flowchart of constructing the health process manifold based on a linear discriminant analysis model combined with Laplacian eigenmaps. **e** Flowchart of calculating the U score of an individual

Actually, assuming there is a health potential on each individual who is at a non-equilibrium steady state from a dynamical systems viewpoint, the individual health status or health potential can be decomposed into two features, i.e. (1) phenotypic state/potential and (2) homeostatic resilience/potential, based on diffusion map theory. In other words, the health status of an individual can be viewed as a function of not only homeostatic potential but also phenotypic state described by a variety of factors and stressors. Here, to quantify individual status with such two features, we developed a new method called health state manifold (HSM) by exploring dynamical information of time-course omics data based on dynamic network biomarker (DNB) method (Chen et al. 2012; Gao et al. 2022; Yang et al. 2018; Zhang et al. 2021) and diffusion map theory. The "homeostatic resilience" indicates a potential or ability to depict the stability or variability for an individual in the current health state or phenotype, while "phenotypic state" represents a potential or state of an individual on the process from health to disease. Thus, HSM can visualize not only the position of current phenotype/state on the health state manifold but also the ability to maintain stability at the current phenotype (state) for each individual (Fig. 1a, c). Specifically, from a computational viewpoint, homeostatic potential of an individual is characterized by the minimum principal curvature from time-course omics data based on DNB method, which actually represents the homeostatic resilience (Fig. 1e). The larger the curvature, the higher the homeostatic potential value, and the better the ability of the individual to maintain the stability at the current state, vice versa. On the other hand, phenotypic state of an individual is characterized as a state on health process manifold or health state manifold (Fig. 1a), which is evaluated by linear discriminant analysis with Laplacian eigenmaps based on multi-omics data. Hence, this method can quantify the health states or distinguish health differences not only for the individuals with a similar phenotype by (1) homeostatic resilience, but also for the individuals with a similar homeostatic resilience by (2) health state manifold (Fig. 1c). To verify our method, we applied HSM method to diabetes mellitus (rat subjects) and the Roux-en-Y Gastric Bypass (human subjects) for constructing their health state manifolds during disease progression process and also recovery process, which demonstrates the effectiveness on the personalized health quantification.

## Methods

### Health Status of an Individual can be Characterized by Phenotypic Potential and Homeostatic Potential

From a dynamical systems viewpoint, health state ${\boldsymbol{x}}(t)$ (omics data, e.g., a gene expression vector or a protein expression vector) of an individual dynamically changes with time and condition, and can be represented by a dynamical system, i.e. a stochastic dynamical system with a birth–death term (Shi et al. 2022, 2019; Wang et al. 2011; Weinreb et al. 2018). As the available data, the state ${\boldsymbol{x}}(t)$ is observed for each individual. Assuming there is a health potential representing the health state of an individual which is considered as a non-equilibrium steady state, then the health potential $F({\boldsymbol{x}}(t))$ of the individual can be decomposed into (1) phenotypic state/potential and (2) its homeostatic resilience/potential based on the diffusion map theory and continuous birth–death process (Shi et al. 2019; Wang et al. 2011; Weinreb et al. 2018). Thus as shown in Fig. 1,

1. we use $V({\boldsymbol{x}}\left(t\right))$ score to characterize phenotypic state of an individual, which is evaluated by linear discriminant analysis with Laplacian eigenmaps, and
2. we use $U({\boldsymbol{x}}(t))$ score to characterize homeostatic potential of the individual, which is estimated by the minimum principal curvature from time-course omics data based on DNB method (Fig. 1a, b).

Hence, the health status/state of an individual is described by both $V$ score and $U$ score together based on the observed data ${\boldsymbol{x}}$ at each stage or time $t$, whose computational procedures are given next in details.

### Characterizing Phenotypic Potential ${\boldsymbol{V}}$ and Constructing Health Process Manifold

To estimate $V$ that characterizes phenotypic state/potential of each individual, we applied a linear discriminant analysis model with Laplacian eigenmaps to construct a "health process manifold", visualized as "health state manifold", which is based on another methodology called "health space", described earlier by van den Broek et al. (2017). In this approach, the background group and the target group are used to define health process manifold. This means that the health process manifold is constructed to reflect the differences between the individuals with optimal phenotype from the background group and the individuals with worst phenotype from the target group. The $V$ score and visualization of individuals with various phenotypes or states is based on this predefined health process manifold. The positions/states of individuals on the manifold reflect their current health status. The distance between individuals on the manifold reflects the similarity of their current health status (or phenotypes). The closer individuals are on the manifold, the more similar their health status is. The model was trained to discriminate between background group and target group using a tenfold cross-validated linear discriminant analysis model combined with Laplacian eigenmaps.

### Linear Discriminant Analysis Model Combined with Laplacian Eigenmaps for Characterizing ${\boldsymbol{V}}$

Given a matrix ${X}_{(n+m)\times p}$, that is composed of two groups, $X=\left({X}^{H},{X}^{D}\right)=({x}_{1}^{H},\dots ,{x}_{m}^{H},{x}_{1}^{D},\dots ,{x}_{n}^{D})$, where ${X}^{H}$ is the background group of the individuals with optimal phenotype with $m$ samples and ${X}^{D}$ is the target group of the individuals with worst phenotype with $n$ samples. In addition, both samples ${x}_{i}^{H}$ and ${x}_{j}^{D}$ $(i=1,\dots ,m;j=1,\dots ,n)$ are $p$-dimensional vectors, e.g., $p$ molecular expressions or $p$ variables. However, generally the dimension of $p$ is too high to discriminate and visualize the phenotypes, so we map $X$ to a low-dimension space via Eq. (1):

1 $$\begin{array}{c}Y=XC, s.t.{C}^{T}C=I\end{array},$$

where $Y$ is the projection of $X$ onto a low-dimension space, $C$ is the matrix to be obtained, the superscript $T$ represents matrix transpose, and $I$ is the identity matrix.

One of our goals is to discriminate ${X}^{H}$ and ${X}^{D}$. Therefore, the two groups should be as far away as possible in the low-dimension space after mapping, that is to say, ${Y}^{H}$ and ${Y}^{D}$ should be as far as possible. Consequently, the initial target function is formula (2):

2 $$\begin{array}{c}\underset{C}{\mathrm{max}}tr\left({\left({\overline{Y} }^{D}-{\overline{Y} }^{H}\right)}^{T}\left({\overline{Y} }^{D}-{\overline{Y} }^{H}\right)\right),\end{array}$$

where ${\overline{Y} }^{H}$ is the sample mean of the individuals from the background group and ${\overline{Y} }^{D}$ is the sample mean of the individuals from the target group. The symbol $tr()$ represents the trace of the matrix, which is equal to the sum of all the eigenvalues of the matrix. Combining Eq. (1) and formula (2), it can be deduced that formula (2) is equivalent to formula (3), which is one of the target functions:

3 $$\begin{array}{c}\underset{C}{\mathrm{max}}tr\left({C}^{T}{\left({\overline{X} }^{D}-{\overline{X} }^{H}\right)}^{T}\left({\overline{X} }^{D}-{\overline{X} }^{H}\right)C\right), s.t.{C}^{T}C=I\end{array},$$

The maximum of formula (3) is the sum of $d$ maximum eigenvalues of $B (B={\left({\overline{X} }^{D}-{\overline{X} }^{H}\right)}^{T}\left({\overline{X} }^{D}-{\overline{X} }^{H}\right)$. $d$ is the dimension after dimension reduction. And $C$ is the corresponding eigenvector matrix.

Another goal is to construct a health process manifold, where the positions/states of individuals reflect their current health status. Laplacian eigenmap was applied because of its characteristic that maintains the distance between samples in the low-dimension space after mapping similar to that in original space. The target function of Laplacian eigenmap is as formula (4):

4 $$\begin{array}{c}\underset{C}{\mathrm{min}}\displaystyle\sum_{i,j}{\Vert {y}_{i}-{y}_{j}\Vert }^{2}{W}_{ij}, \left(i,j=1,\dots ,n+m\right),\end{array}$$

where $W$ is the adjacency matrix of $X$ constructed by Pearson correlation coefficients (PCC) and ${y}_{i}$ is the projection of ${x}_{i}$ onto a low-dimension space. After mathematical derivation (Supplemental Materials. derivation), it can be obtained that formula (4) is equivalent to formula (5):

5 $$\begin{array}{c}\underset{C}{\mathrm{min}}tr({Y}^{T}LY), s.t.{Y}^{T}DY=I\end{array},$$

where $L$ is the Laplacian matrix, $L=D-W$, $({D}_{ii}={\sum }_{j=1}^{n+m}{W}_{ij})$, diagonal matrix $D$ is the degree matrix. Combining Eq. (1) and formula (5), it can be deduced that formula (5) is equivalent to formula (6):

6 $$\begin{array}{c}\underset{C}{\mathrm{min}}tr\left({C}^{T}{X}^{T}LXC\right), s.t.{C}^{T}{X}^{T}DXC=I\end{array},$$

The minimum of formula (6) is the sum of $d$ minimum eigenvalues of $\Sigma (\Sigma ={X}^{T}LX)$, $d$ is the dimension after dimension reduction. And the corresponding eigenvector matrix is the $C$ that should to be obtained.

Combining target function (3) and target function (6), our final target function can be obtained as formula (7), which meets both of our goals:

7 $$\begin{array}{c}\mathrm{max}\frac{tr\left({C}^{T}BC\right)}{tr\left({C}^{T}\Sigma C\right)}\end{array},$$

$$s.t. {C}^{T}C=I,$$

$${C}^{T}{X}^{T}DXC=I,$$

where $B={\left({\overline{X} }^{D}-{\overline{X} }^{H}\right)}^{T}\left({\overline{X} }^{D}-{\overline{X} }^{H}\right)$ and $\Sigma ={X}^{T}LX$. The maximum of formula (7) is the sum of $d$ maximum eigenvalues of ${\Sigma }^{-1}B$, and the corresponding eigenvector matrix is the $C$ that should to be obtained.

Substituting the obtained $C$ into Eq. (1), then we obtain $Y$, which is the $V$ scores and the positions on the health process manifold of individuals.

### ${\boldsymbol{V}}$ Score Algorithm

Step1. Input: background data matrix ${X}^{H}\in {\mathbb{R}}^{m\times p}$, target data matrix ${X}^{D}\in {\mathbb{R}}^{m\times p}$, reference data matrix $X=({X}^{H},{X}^{D})\in {\mathbb{R}}^{(m+n)\times p}$, dimension $d$, test data matrix ${X}^{M}\in {\mathbb{R}}^{k\times p}$.

Step2. Compute Laplacian matrix $L$, $\Sigma ={X}^{T}LX$,

Compute $$B={({\overline{X} }^{D}-{\overline{X} }^{H})}^{T}({\overline{X} }^{D}-{\overline{X} }^{H})$$,

Compute $d$ maximum eigenvalues of ${\Sigma }^{-1}B$ and the corresponding eigenvector matrix $C\in {\mathbb{R}}^{p\times d}$,

Compute $$=XC$$ $$\in {\mathbb{R}}^{(m+n)\times d}$$, $${Y}^{M}={X}^{M}C\in {\mathbb{R}}^{k\times d}$$, $${V}_{i}={Y}_{i1}$$.

Step3. Output: each individual's $V$ score ${V}_{1\dots (m+n+k)}$.

### Tenfold Cross-Validation

Given a matrix ${X}_{(n+m)\times p}$, that is composed of two groups, $X=\left({X}^{H},{X}^{D}\right)=({x}_{1}^{H},\dots ,{x}_{m}^{H},{x}_{1}^{D},\dots ,{x}_{n}^{D})$, where ${X}^{H}$ is the background group with $m$ samples and ${X}^{D}$ is the target group with $n$ samples. Dividing $X$ randomly into ten parts, we take nine parts as the training set to train the model of health process manifold and another as the test set. In this way, the method is repeated ten times in such a way that each individual has a 90% chance of being in the training or in the test set, and each time we got coefficients of each feature. Then we calculated the coefficient of variation ($cv$) of the ten coefficients of each feature from the ten models trained through $cv=sd/mean$. The features with small $cv$ (about the 70 percent) are considered as stable features and are screened out for training the model of health process manifold. The flowchart of tenfold cross-validation was shown in SI. Fig. S8.

### Characterizing Individual Homeostatic Potential ${\boldsymbol{U}}$

As described above, health status of an individual includes two parts, i.e., state (phenotype) and its stability (resilience) for this individual. Thus, quantifying and evaluating health status should consider the homeostatic potential of individuals, in addition to their phenotypes or states. Therefore, how to evaluate the homeostatic potential or the tipping point of a biological process is an important problem (Chen et al. 2012). To solve it we introduce curvature from differential geometry, which is used to measure how curved the surface is. The minimum principal curvature of an individual's homeostatic potential landscape constructed with molecular expression from at least three time points is applied to characterize the homeostatic potential of an individual. Individuals with high homeostatic potential tend to maintain their current stable state under perturbations, while individuals with small homeostatic potential tend to transfer to another state and correspond to the tipping point.

Given an individual $s$, ${X}_{g\times n}$ is molecular expression data of individual $s$ from at least three time points in a same state/stage, where $g$ is the number of molecules and $n$ is the number of samples ($n\ge 3$). The homeostatic potential landscape is constructed according to Eq. (8) (Li and Wang 2013; Wang et al. 2010a, 2008, 2010b):

8 $$\begin{array}{c}U\left({\boldsymbol{x}}\right)=-log P\left({\boldsymbol{x}}\right),\end{array}$$

where ${\boldsymbol{x}}=({{\boldsymbol{x}}}_{1},\dots ,{{\boldsymbol{x}}}_{g})$ and ${{\boldsymbol{x}}}_{g}$ is a vector of molecules in terms of expression at multiple time points. $P({\boldsymbol{x}})$ represents the probability distribution of the molecular state ${\boldsymbol{x}}$. $U\left({\boldsymbol{x}}\right)$ is the homeostatic potential landscape of the individual. For the simplification of calculating, we define $P({\boldsymbol{x}})$ as a multivariate normal distribution. The calculation of joint probability distribution $P({\boldsymbol{x}})$ is often difficult in reality because of the high dimension $g$ of $X$, so that it is necessary to reduce the dimension of $X$. We assume that the genes are independent of each other. Then we obtain the Eq. (9):

9 $$\begin{array}{c}U\left({x}_{1},{x}_{2},\dots ,{x}_{g}\right)=-logP\left({x}_{1},{x}_{2},\dots ,{x}_{g}\right) =-\left[logP\left({x}_{1}\right)+logP\left({x}_{2}\right)+\dots +logP\left({x}_{g}\right)\right]\end{array}.$$

However, in fact, most genes are related to each other to a certain extent. In order to reduce the loss of information about inter-gene regulation network caused by the above hypothesis, we have partially adopted the idea of landscape DNB (l-DNB) (Liu et al. 2019). The l-DNB method evaluates the local score gene by gene and then compiles the overall local scores into a landscape. The global score can then be calculated from the landscape of the sample by choosing those genes with the highest local scores. For gene $i$ (other molecules are the same as genes), a gene coexpressed/regulatory network is constructed with Pearson correlation coefficient (PCC) and then the first-order neighbors of gene $i$ are selected as the local module for gene $i$. Then principal component analysis (PCA) is applied on the local module, and the mean vector and covariance matrix $(\mu ,\Sigma )$ of the data after dimension reduction are calculated as the parameters of the multivariate normal distribution $P({\boldsymbol{x}})$, so that the homeostatic potential landscape $U({\boldsymbol{x}})$ is obtained by Eq. (8). The minimum principal curvature of $U({\boldsymbol{x}})$ is calculated as the local homeostatic potential value of gene $i$, i.e. $U$ score. In particular, the $U$ score is equal to the minimum eigenvalue of Hessian matrix of $U\left({\boldsymbol{x}}\right)$ when $U\left({\boldsymbol{x}}\right)$ is a function of two variables. Again, the local $U$ score for every gene can be calculated. All of genes are sorted by ascending score. The individual’s global $U$ score can then be calculated by averaging value over top-$k$ genes in the sorted list which lowers the individual’s stability. Individuals with high global $U$ score tend to maintain their current stable state under perturbations, while individuals with small global $U$ score tend to transfer to another state, which actually corresponds to the tipping point or critical state before the transition.

### $U$ Score Algorithm

Step1. Input: molecular expression data matrix $X\in {\mathbb{R}}^{g\times n}$ of individual $s$.

Step2. For each molecule $i$:

Compute the first-order neighbors of molecule $i$ with PCC, molecule $i$’s local module ${X}^{(i)}=({X}_{i},{X}_{neighbors})$,

Compute ${Y}^{(i)}=(PC1,PC2)\in {\mathbb{R}}^{2\times n}$ of ${X}^{(i)}$ with PCA, mean vector and covariance matrix ($\mu ,\Sigma$) of ${Y}^{(i)}$,

Compute bivariate normal distribution $P\left(x\right)$ with ($\mu ,\Sigma$), $U\left(x\right)=-logP(x)$,

Compute the minimum eigenvalue ${\lambda }^{(i)}=\lambda (Hessian(U(x){|}_{x=\mu }))$.

Step3. Compute ${U}_{s}$ by averaging value over top-$k$ molecules sorted by ascending $\lambda$.

Step4. Output: the homeostatic potential ${U}_{s}$ for individual $s$.

### HSM Algorithm

The HSM algorithm is given as follows.

*Step1. Input data*: Input the reference data $X=\left({X}^{H},{X}^{D}\right)$ with omics data of the individuals of background group ${X}^{H}$ (*m* samples) and target group ${X}^{D}$ ($n$ samples) for characterizing $V$; input the time-course or stage-wise data ${X}_{g\times n}$ of each individual for characterizing $U$.

*Step2. *$V$* score*: Calculate the health process manifold with $X=\left({X}^{H},{X}^{D}\right)$ using LDA-LE (formula  7), and then estimate each sample's $V$ score by the regression value of the manifold model (Eq. 1) (*V* score algorithm).

*Step3. *$U$* score*: Calculate the homeostatic potential landscape $U(x)$ with ${X}_{g\times n}$ by Eq. 8 and then estimate the minimum eigenvalue of Hessian matrix of $U(x)$, for each gene $i$ of an individual. Finally, calculate the individual’s global $U$ score by averaging value over top-$k$ genes in the sorted list with Eq. 9 (*U* score algorithm).

*Step4. Output data*: Output the results of $V$ and $U$ scores for each individual, and present them as an HSM against various features.

## Results

### Decomposing Health Status of an Individual into Phenotypic State and its Homeostatic Resilience by Health State Manifold

Traditional approach to evaluate individual health was based on the static biomarkers and clinical features, such as BMI and plasma glucose. It performed well in describing individuals with phenotypic differences, i.e., effective for phenotypic state, but was less effective in describing individuals with similar phenotypes but different deterioration risk, i.e., less effective for homeostatic resilience/potential. For instance, for the individuals with similar phenotypes/states, some with high resilience could maintain relative stability in the current state when disturbed, while others with low resilience have poor ability to maintain a relatively stable state and are prone to transfer into other deteriorated states when disturbed (Fig. 1b). Thus, both phenotypic state and homeostatic potential are necessary components to comprehensively and accurately quantify individual health.

From a dynamical systems viewpoint, health status of an individual dynamically changes with time and condition, and can be represented by a dynamical system. It is a multi-stage process from health to disease state with gradual state change in each stage but occasionally a drastic state transition between two stages (Fig. 1a). Assuming there is a health potential representing the health status of an individual who is at a non-equilibrium steady state from a dynamical systems viewpoint, then the health potential of the individual can be decomposed into (1) phenotypic state/potential and (2) its homeostatic resilience/potential based on the diffusion map theory and continuous birth–death process (Shi et al. 2019; Wang et al. 2011; Weinreb et al. 2018). To quantify individual health, the health status of an individual is decomposed into phenotypic state/potential which is represented by $V$ score and homeostatic potential which is represented by $U$ score. The $V$ score characterizes the current phenotype or state on the whole health process of an individual (Fig. 1a), while the $U$ score explains the ability of an individual to maintain relative stability at this state (Fig. 1b). Hence, an individual’s health can be described by $V$ and $U$ together (Fig. 1a, c). This approach to numerically characterize phenotypic state/potential and homeostatic potential with $V$ score and $U$ score respectively is named HSM method.

To characterize phenotypic state/potential, we developed a method, i.e., linear discriminant analysis with Laplacian eigenmap (LDA-LE) to construct a health process manifold based on omics data and phenotypic data of a population including the individuals with optimal phenotype and the individuals with worst phenotype. Specifically, high-dimensional omics data and phenotypic data of each individual is reduced to low dimensions space by linear discriminant analysis with Laplacian eigenmap to construct a health process manifold, and then we numerically characterize the phenotype by the position or relative distances between the current state of each individual and the disease state on the manifold, i.e., the $V$ score (Fig. 1d), rather than directly calculate the phenotype. On the other hand, to characterize homeostatic potential, we represent the homeostatic potential of an individual by $U\left({\boldsymbol{x}}\right)=-\text{log }P\left({\boldsymbol{x}}\right)$ (Li and Wang 2013; Wang et al. 2010a, 2008, 2010b). Here, ${\boldsymbol{x}}$ is a state/vector of the measured variables (e.g. omics data), and $P({\boldsymbol{x}})$ represents the probability distribution at the state${\boldsymbol{x}}$. Then we can use the minimum principal curvature of $({\boldsymbol{x}})$, i.e., the $U$ score, to characterize the resilience ability of the individual at the state ${\boldsymbol{x}}$ (Fig. 1e), rather than directly calculate the homeostatic potential. The individuals with large curvature have high resilience ability, while the individuals with small curvature have low resilience ability.

### Quantifying Health State by HSM on Rats Diabetes Dataset

The GSE13270 dataset from Gene Expression Omnibus (GEO) database (https://www.ncbi.nlm.nih.gov/geo/) is a time series data for the type 2 diabetes progression and the development of insulin resistance in two animal models: the GotoKakizake (GK) rat and the WistarKyoto (WKY) rat, with and without a high fat diet superimposed on the two models. In the dataset, 50 liver samples from GK rats and 51 liver samples from WKY rats were fed with normal diet (ND) and high fat diet (HFD) (SI. Table 3). The gene expression profiles were tested at five different rat ages: 4, 8, 12, 16, and 20 weeks. First, to reduce the impact from other tissues and pathways (noise), we screened 362 genes of rat species associated with type 2 diabetes by the National Center for Biotechnology Information (NCBI) database (https://www.ncbi.nlm.nih.gov/gene) and used these 362 genes for the following analysis (SI. Table 1). The data of each gene used for the analyses were normalized with z-score normalization.

**Fig. 2:**
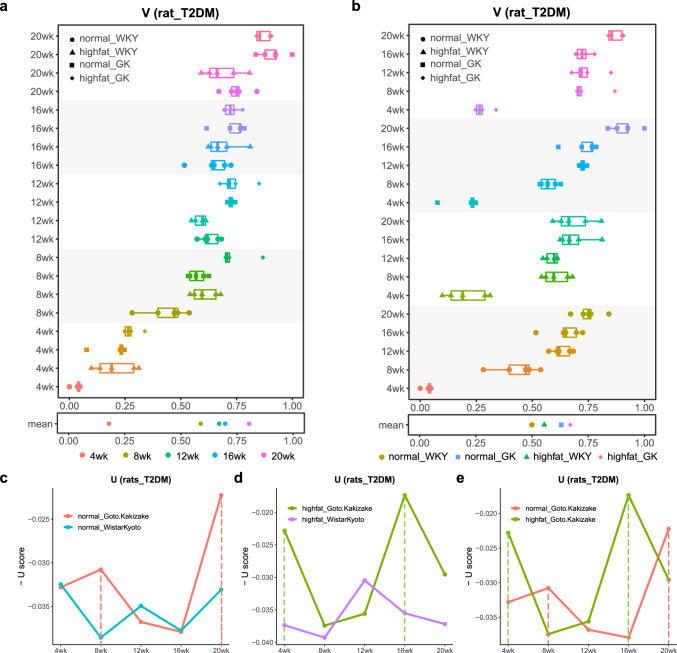
V and U of the rats diabetes dataset. **a** health process manifold sorted by age. The V scores increased with age. At the same age, WKY rats were closer to background group than GK rats. **b** health process manifold sorted by rat type and dietary pattern. Rats with normal diet were closer to background group than rats with high fat diet. With the increase of age, the rats were closer to target group. **C** GK rats fed with normal diet had low-potential and high transfer risk at the age of 8 weeks and 20 weeks. **d** GK rats fed with high fat diet had low-potential and high transfer risk at the age of 4 weeks and 16 weeks. **e** Compared with GK rats with normal diet, the two low-potential points of GK rats with high-fat diet were all one stage ahead

To estimate $V$ score that characterizes a phenotype, the WKY rats at the age of four weeks fed with normal diet were chosen as the background group (the group with optimal phenotype) and the GK rats at the age of 20 weeks fed with high fat diet were chosen as the target group (the group with worst phenotype) to define a health process manifold. Each sample in the two groups was mapped to a low-dimensional system of coordinate (Fig. 1d), the samples of background group were artificially assigned to a high score (mapped to around of the maximum in the coordinate system) and the samples of target group were artificially assigned to a low score (mapped to around of the minimum in the coordinate system), and the position of each sample in the coordinate system was defined as the $V$ score of the sample (Fig. 1d). The position of other samples on the health process manifold was based on the regression values of the health process manifold model, which reflected the sample's current health status (Fig. 1d). In the following figures, in order to show the health process from health to disease more clearly, we do not directly show the $V$ score of each individual, but show the $-V$ score of each individual, i.e., the samples of background group were around of minimum in the coordinate system and the samples of target group were around of maximum in the coordinate system. The $V$ scores of all samples were calculated and shown in Fig. 2a based on groups, and the x axis was the $-V$ score of each sample (Fig. 2a). The y axis was 20 groups sorted by rat age (Fig. 2a). This sort clearly showed the change of $V$ scores with increasing age. The samples with near positions on the manifold had similar health states. The four groups at the rat age of four weeks were positioned between 0 and 0.34 on the x-axis with mean value of 0.18 (Fig. 2a), the groups at the age of 20 weeks were positioned between 0.59 and 1 on the x-axis with mean value of 0.8 (Fig. 2a), and the mean values for the groups at the age of 8, 12, 16 weeks were 0.59, 0.67, 0.7 (Fig. 2a), respectively. The *t* tests were calculated for pairwise comparisons among the five groups at different ages. All the *p* values were less than 0.05 except that between group at 12 weeks and group at 16 weeks. In the 12- and 16-week-old samples, the $V$ scores of WKY rats were significantly smaller than those of GK rats (*t*-test, *p*-value = 1.403e−05). These indicated that the health states of rats at different ages were obviously different, and the state of WKY rats was closer to background group than the GK rats at same age. The samples were also divided into four sets by rat type and dietary pattern: WKY rats with normal diet, WKY rats with high fat diet, GK rats with normal diet and GK rats with high fat diet. Each set contained the five groups of different ages (Fig. 2b). This sort clearly showed the difference in $V$ scores between samples with different rat types and dietary patterns. On this manifold, the means of $-V$ scores of the four sets were 0.5, 0.55, 0.63 and 0.67, respectively. And in each set, the $V$ scores of the samples with the same rat type and the same dietary pattern are changed with age increasing (Fig. 2b). This indicated that the $V$ scores can describe the health process of rats from health state to disease state. And individuals at different states can be distinguished by $V$ scores.

On the other hand, to calculated $U$ score that characterizes homeostatic potential, multi-samples data are required. Hence, we calculated the potential $U$ score based on all samples of each group. After calculating the $U$ score at each time point of rat age, we found that there were two time points with low homeostatic potential for the GK rats in the disease development of T2DM compared with the WKY rats (Fig. 2c). For the GK rats with normal diet, strong signal of potential first appeared at the age of eight weeks and then at 20 weeks, again (Fig. 2c). Actually, these low homeostatic potential periods were also consistent with the development of T2DM in GK rats, according to the plasma glucose and the plasma insulin of concentrations from GK and WKY rats at different ages (Almon et al. 2009). Some studies have described some significant differences during development between the GK rats and the WKY rats in the terms of weight, plasma glucose and plasma insulins (Almon et al. 2009). Specifically, the WKY rats gained significantly more weight than the GK rats since the age of eight weeks (Almon et al. 2009). Plasma insulin in GK rats increased sharply between four and eight weeks, began to decline after eight weeks, and GK concentrations were lower than WKY at 20 weeks, suggesting beta-cell failure (Almon et al. 2009). These observations revealed two key time points of the state transition, consistent with the results of $U$ scores. These suggested that the $U$ score is effective for characterizing homeostatic resilience, which is able to distinguish individuals with similar phenotypic state and show strong signal at critical points. For the GK rats with high fat diet, the signals appeared ahead of the GK rats with normal diet (Fig. 2e), this is, the signal of homeostatic potential appeared at the age of four weeks and then again at 16 weeks (Fig. 2d). The high risk of state transition for the GK rats with normal diet appeared at eight and 20 weeks, and for the GK rats with high fat diet appeared at four and 16 weeks. This means that high fat diet may have accelerated the onset of T2DM.

Combining $V$ and $U$, we successfully quantified individual health of rats. The different phenotypic states in the development of the rats T2DM were distinguished by $V$ score, while $U$ score distinguished individuals at similar phenotypic states. For example, the 12-week-old GK rats with high fat diet and the 16-week-old GK rats with high fat diet had similar $V$ scores (Fig. 2a), that is, they had similar phenotypic state. But the different $U$ scores can distinguish the two groups (Fig. 2d). The GK rats at 12 weeks were stable in the current state, while the GK rats at 16 weeks were at critical point. Correspondingly, GK at 12 weeks with normal diet and WKY at 12 weeks with normal diet had similar $U$ scores, indicating that they were both stable in the current state, but they had significant difference in $V$ scores (*t* test, *p* value = 0.007). By using the two indexes, we can distinguish individuals that are difficult to be distinguished by a single index, so that we can quantify individual health more accurately, which will contribute to early warning and intervention of diseases.

### Quantifying Health by HSM for Human Roux-en-Y Gastric Bypass Dataset

The HSM method can describe the individual health state in the process of not only development from health to disease, but also recovery from disease to health. Applying the approach to a dataset comprises the time series data of 19 individuals' recovery process after Roux-en-Y Gastric Bypass (RYGB) surgery. In the dataset, the plasma samples were collected from nine normal glucose-tolerant obese (NO) and 10 diabetic obese (DO) patients before and 1-week, 3-months, 1-year after RYGB, and the plasma samples were obtained at three time points for each individual, including the fasting state and 30 min and 45 min after a standardized liquid meal (Li et al. 2018) (SI. Table 4). One-hundred and twenty-eight metabolites were measured from both NO and DO samples at all four phases. The data of each gene used for the analyses were normalized with z score normalization.

**Fig. 3:**
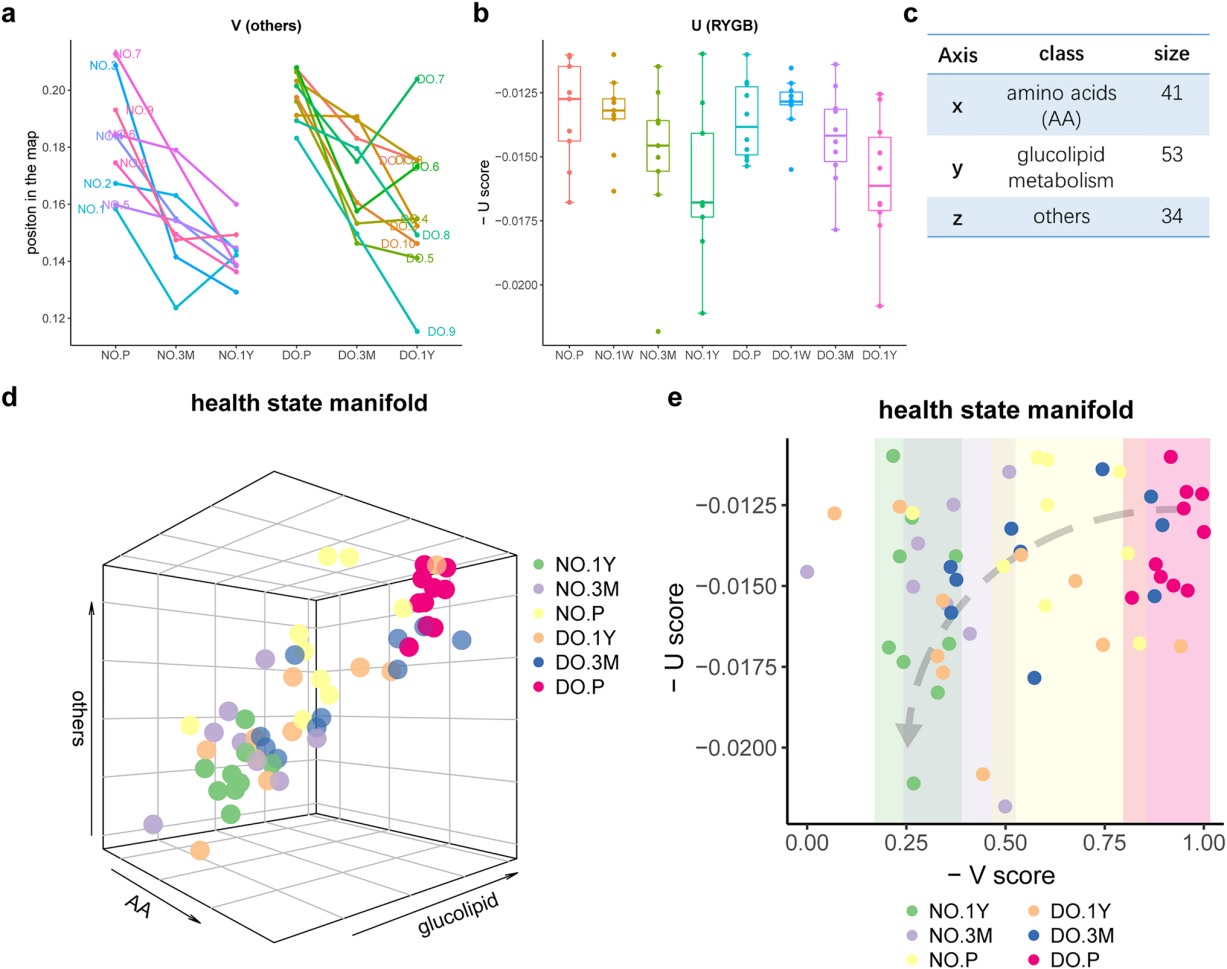
V and U of the RYBG dataset. **a** Individual's positions in the "others"-axis (– V scores) at the before, 3-months and 1-year after RYGB stages decreased gradually with recovery time. **b** The – U score of every individual at each stage. For both NO and DO group, the median of the potential values of each group gradually improved from before surgery to 1-year after surgery, and the risk of state transfer decreased gradually. **c** The 128 metabolites were divided into three classes: amino acids (41), glucolipid metabolism (53) and others (34) as the three axes of the health state manifold. **d** Health state manifold shows the V score characterized the phenotype of every individual at each stage. **e** The health state of each individual before and after the Roux-en-Y Gastric Bypass surgery was described by V and U. And clearly populations can be distinguished by V and U

First, the health process manifold that revealed the process of recovery from before surgery to 1-year after surgery was constructed, and the state of each individual at each stage was obtained by the position on the manifold. The NO group at 1-year (NO.1Y) after surgery was chosen as the background group (group with optimal phenotype) and the DO group before surgery (DO.P) was chosen as the target group (group with worst phenotype), and then the model of the manifold was defined by the two groups. The $V$ score of every individual at all four phases was calculated based on the regression values of the manifold model. The health state manifold is a visualization of $V$ scores of individuals (Fig. 3d). In particular, 1-week after RYGB was considered as the most unstable stage of recovery, which is consistent with the following $U$ scores (Fig. 3b). Therefore, the samples in 1-week group were not shown on the health state manifold. The 128 metabolites were divided into three classes: amino acids (41 molecules), glucolipid metabolism (53 molecules) and others (34 molecules) as the three axes of the health state manifold (Fig. 3c, d). Specifically, only the values of fasting state of the factors were used to define the health process manifold and calculate the $V$ scores in each class. The $-V$ scores of all individuals were shown in Fig. 3d. For an individual, its $-V$ scores at the before, 3-months and 1-year after RYGB stages decreased gradually in all three axes with recovery time (Fig. 3d), this is, the $V$ scores increased gradually with recovery time, the $-V$ scores on each axis can be shown in Figs. 3a and SI. On the health state manifold, the position of the NO group before surgery (NO.P) on the health state manifold is close to the DO.P group (the worst healthy group), and for the DO group, their position on the map gradually approached that of the NO.1Y group (background group) with postoperative recovery time, consistent with trends in their BMI and fasting plasma glucose (Figs. 3d and S2), indicating that $V$ scores can describe different phenotypic states of individual in the recovery process.

As mentioned before, health potential is made up of two parts. Except for the $V$ score of each individual, the $U$ was also calculated. For both NO and DO group, the median of the $U$ scores of each group gradually improved from before surgery to 1-year after surgery, and the risk of state transition decreased gradually (Fig. 3b). The NO.P and DO.P groups had the lowest $U$ scores and the highest risk of transition (Fig. 3b). The NO.1Y and DO.1Y groups had the highest $U$ scores and the lowest state-transition risk among the four stages (Fig. 3b). And the homeostatic potential of each individual changed with postoperative recovery time in line with the changes in their BMI and plasma glucose (SI. Fig. S2). It should be noted that the risk of state transition was slightly higher at 1-week after RYGB than before-surgery (Fig. 3b), possibly indicating that 1-week after RYGB was a very unstable stage of recovery. To validate the homeostatic potential on describing individuals at the same stage, which was similar in phenotypes, individuals at the same stage were divided into two groups by the mean of $U$ scores. Those larger than the mean was considered as the high-potential group (high-stability group or low-transfer-risk group), while those smaller than the mean was regarded as the low-potential group (low-stability group or high-transition-risk group). Then the significance of the difference between the phenotypes of the two groups was tested by *t* test, and the results showed that the BMI of the high-risk group was significantly higher than that of the low-risk group at NO.P, NO.1Y and DO.3M stages (Table 1), and the difference in fasting plasma glucose between the two groups was significant at the NO.1Y stage, the high-risk group had higher fasting plasma glucose (*p* value < 0.05) (Table 1). These indirectly supported that the $U$ score can distinguish individuals with similar phenotypic state and detect critical points.

**Table 1:** Difference of baseline between the high-risk group and the low-risk group

		Diabetic obesity (10 DO subjects)			Normoglycemic obesity (9 NO subjects)		
		Mean_high	Mean_low	*p*-value	Mean_high	Mean_low	*p*-value
Pre	BMI	44.663	41.294	0.118	43.863	38.796	0.070(*)
	glu	9.633	8.680	0.289	5.287	5.792	0.157
1W	BMI	43.198	40.155	0.135	41.249	40.058	0.388
	glu	7.256	6.658	0.221	4.980	5.258	0.264
3M	BMI	39.445	35.569	0.076(*)	33.800	35.863	0.242
	glu	6.273	7.513	0.144	5.075	5.025	0.416
1Y	BMI	33.798	33.687	0.488	34.837	29.481	0.054(*)
	glu	5.799	6.752	0.219	5.118	4.921	0.044(**)

Individuals at the same stage were divided into two groups by the mean of U scores. The "mean_high" represents the mean of baseline (BMI and glu) of the group with high-transition-risk, and the "mean_low" represents the mean of baseline of the group with low-transition-risk. The "*p*-value" represents the *p*-value of *t*-test between the baseline of the two groups at each stage. The results suggested that the BMI of the high-risk group was significantly higher than that of the low-risk group at NO.P, NO.1Y and DO.3 M stages. And the glu of the high-risk group was significantly higher than that of the low-risk group at NO.1Y

Combining $V$ and $U$, the health state manifold that reflects both phenotypic state/potential and homeostatic potential was visualized (the $V$ score in Fig. 3e was calculated from $V$ score of the three dimensions in Fig. 3d by Principal Component Analysis) (Fig. 3e). The health status of an individual was quantified and described by $V$ and $U$. The $V$ score characterized phenotypic state/potential and represented the current position of the individual in the whole health process while the $U$ score characterized homeostatic potential and represented the stability of the individual in the current position/state (Fig. 3e). The results indicated the health difference not only for the individuals with similar phenotypic state/potential by homeostatic potential, but also for the individuals with similar homeostatic potential by phenotypic state/potential in health state manifold (Fig. 3e). For example, the individuals of the NO.1Y group had similar $V$ scores, but their $U$ scores fluctuated greatly (Fig. 3e). And the individuals with low-potential had significantly higher BMI than those with high-potential (*p* value = 0.05) (Table 1). This indicated that individuals with low-homeostatic potential were closer to the critical point, despite similar $V$ scores. Correspondingly, although the $U$ scores of NO.P group and DO.P group were similar, the $V$ scores between the two groups were different significantly (*p*-value = 0.0007) (Fig. 3e).

### Health Detection for Human Hepatocellular Carcinoma Dataset

The GSE6764 dataset comprises the data of the stepwise carcinogenic process from pre-neoplastic lesions to hepatocellular carcinoma (HCC) covering six stages and was obtained from the GEO database (https://www.ncbi.nlm.nih.gov/geo/) to be used to evaluate health status at each stage. The six stages were cirrhotic, dysplastic, very early HCC, early HCC, advanced HCC and very advanced HCC. In the dataset, 75 human liver tissue samples covering six different stages with at least seven samples at each stage were collected and examined the gene expression profiles of them (SI. Table 5). There were 178 HCC-related genes to be identified from the NCBI database and used for the following analysis (SI. Table 1). The data of each gene used for the analyses were normalized with z score normalization.

**Fig. 4:**
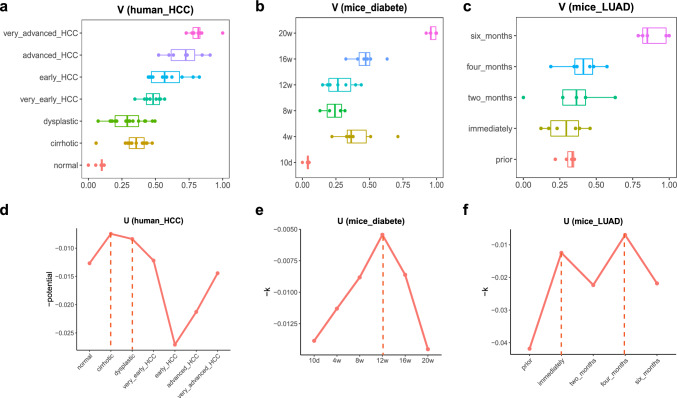
V and U of three other datasets. **a–c** The –V scores of samples at every stage in the HCC dataset, mice diabetes dataset and the LUAD dataset, respectively. **d–f** The –U scores of the samples at every stage in the HCC dataset, mice diabetes dataset and the LUAD dataset, respectively

First, to estimate $V$ score that characterizes phenotype, the normal samples and the samples with very advanced HCC were chosen as the background group and the target group respectively, and they were used to define the health process manifold model. The $V$ score of every sample was calculated based on the regression values of the manifold model. On the health process manifold, the y axis was the different stages from normal to very advanced HCC, and x axis was the $-V$ scores of samples (Fig. 4a). The normal group was at the minimum value of the x axis, the very advanced HCC group was at the maximum value of the x-axis. The $-V$ scores of the other groups gradually increased from the minimum value to the maximum (i.e., $V$ scores gradually decreased from the maximum to the minimum), which was consistent with the order of the development of the disease (from the cirrhotic state to the advanced HCC state) (Borzio et al. 1995; Ho et al. 1981; Kew and Popper 1984). Particularly, the $V$ scores of samples between cirrhotic stage and dysplastic stage were close to each other (Fig. 4a), and both of them are in precancerous stages. This might be due to the fact that cirrhosis was usually accompanied with dysplasia (Ho et al. 1981). Among the four cancer stages (from the very early HCC stage to the very advanced HCC stage), the $V$ scores of samples at different stages were obvious different (*p* value of *t* test between $V$ scores of samples at very early HCC stage and samples at early HCC stages is 0.02, *p* value between samples at early HCC stages and advanced HCC stage is 0.05 and *p*-value between samples at advanced HCC stage and very advanced HCC stage is 0.04.) (Fig. 4a). On the other hand, the homeostatic potential landscape was constructed at each stage or state, whose $U$ score was calculated to explain the state stability at each stage (Fig. 4d). We found that the $U$ score was low in the cirrhosis stage and the dysplasia stage, indicating that individuals at the two stages had low stability and high risk of state transition, which was consistent with previous reports that cirrhosis and liver cell dysplasia were two major risk factors for HCC (Borzio et al. 1995; Kew and Popper 1984). Combining $V$ and $U$, we can distinguish the individuals with similar phenotypes by $U$ score and distinguish the individuals with similar homeostatic potential by $V$ score.

### Health Detection for Mice Diabetes Dataset

The GSE15150 dataset comprises the time series data of age-independent changes of gene expression in pancreatic lymph nodes (PLNs) during disease induction and progression in non-obese diabetic (NOD) mice. The PLNs were obtained from female NOD mice at six different time points (10 days (seven mice), and four weeks (six mice), eight weeks (four mice), 12 weeks (seven mice), 16 weeks (six mice), and 20 weeks of age (five mice)) and prepared for mRNA analysis (SI. Table 6). There are 875 genes related to non-obese diabetic to be screened from the NCBI database and used for the following analysis (SI. Table 1). The data of each gene used for the analyses were normalized with z score normalization.

First, the health process manifold model was defined by two groups of samples, one was the samples at the age of 10 days (the samples with optimal phenotype) chosen as the background group, and another was the samples at the age of 20 weeks (the samples with worst phenotype) chosen as the target group. The $V$ score that characterizes phenotype of every sample was calculated based on the regression values of the manifold model. On the health process manifold, the y-axis was the different ages from 10 days to 20 weeks, and x-axis was the $-V$ scores of samples (Fig. 4b). The background group (aged 10 days) was at the minimum of the x-axis, the target group (aged 20 weeks) was at the maximum of the x-axis, and the $-V$ scores of the other four groups were located between the two groups, forming a health process manifold. The position of each sample on the health process manifold reflected the current health status of this sample. The $-V$ scores of the groups before 12 weeks of mouse age were closer to the background group than the target group, which was consistent with the reports of public reference and some biological experiments about the development of type 1 diabetes (T1D) in female NOD mice (Anderson and Bluestone 2005; Delovitch and Singh 1997; Green et al. 2002; Kodama et al. 2008). Disease onset of diabetes usually occurs at 12–14 weeks of mouse age in female NOD mice (Anderson and Bluestone 2005), and destructive insulitis, leading to overt hyperglycemia, occurs around 12 weeks of mouse age or later (Delovitch and Singh 1997; Green et al. 2002; Kodama et al. 2008). Specifically, the median of $-V$ scores of the three groups aged eight weeks, 12 weeks and 16 weeks gradually increased (Fig. 4b), consistent with the development of the disease, while the median of $-V$ scores of the 4-week-old group was larger than that of the 12-week-old group (Fig. 4b). It may be due to the reported occurrence of the T cell initiation and lymphocytic infiltration in the 4 weeks of age for female NOD mice (Kodama et al. 2008), and the infiltration of the immune cells surround the islet (peri-insulitis) at approximately three to four weeks of age in NOD mice (Anderson and Bluestone 2005; Delovitch and Singh 1997). On the other hand, the homeostatic potential landscape was constructed and the $U$ scores that characterize homeostatic potential were calculated at each age (Fig. 4e). We found that the $U$ score of the group aged 12 weeks was the lowest among the six stages from 10 days of age to 20 weeks of age, and it is consistent with the reports that disease onset of diabetes usually occurs at 12–14 weeks of mouse age in female NOD mice (Anderson and Bluestone 2005).

Combining $V$ and $U$, although the $V$ scores of the group aged 12 weeks was not close to the target group, the group aged 12 weeks was already at the critical point according to $U$ scores. And although the $U$ scores of the group aged 10 day and the group aged 20 weeks were similar, the $V$ scores of the two groups were significantly different (*p* value = 4.311e−09). These indicated that HSM method can quantify individual health more accurately with $V$ and $U$.

### Health Detection for Mice Lung Adenocarcinoma Dataset

The GSE102707 dataset comprises the time series data of airway gene expression changes prior to lung cancer onset in mice with knockout of the *Gprc5a* gene and tobacco carcinogen (nicotine-specific nitrosamine ketone (NNK)) exposure and that develop the most common type of lung cancer, lung adenocarcinoma (LUAD). Cytologically-normal airway epithelial brushings were collected before exposure (prior), immediately (t0) and every two months following NNK exposure (two, four, and six months) until time of LUAD development (six months), and then analyzed by RNA-sequencing (SI. Table 7). In total, airway brushings from 28 mice were sequenced, and 201 genes related to LUAD were screened from the NCBI database (SI. Table 1). The data of each gene used for the analyses were normalized with z-score normalization.

First, we chose the prior group as the background group and the six months group as the target group to define the health process manifold model. The $V$ score characterizing the phenotype of every sample was calculated based on the regression values of the manifold model. On the health process manifold, the y-axis was the different time points from prior to six months following exposure, and x-axis was the $-V$ scores of samples (Fig. 4c). The position of the sample on the health process manifold reflected the current health status of the sample. The mean of $-V$ scores of the prior group and the immediately group were all around 0.3, and the $-V$ scores of the samples in the two months, four months and six months group were around 0.34, 0.4 and 0.89, respectively, forming a health process manifold from health to onset of disease. Specifically, the position of the samples at the first four stages (prior, immediately, two months, four months) was close and the four groups were far from the group at the fifth stage (six months). It indicates that the phenotypic state at first four stages were relatively close and quite different from the fifth stage, which was consistent with the process of LUAD in mice (Kantrowitz et al. 2018). The six months following exposure is the onset of LUAD, and the first four stages belongs to the range of health (Kantrowitz et al. 2018). On the other hand, the homeostatic potential landscape was constructed and the $U$ scores were calculated at each time point (Fig. 4f). We found there were two low-potential time points, the first low-potential time point was immediately group, which was the time point that following the NNK exposure. It was reasonable for mice to be in unstable state (i.e., critical state or pre-disease state) because the mice might begin to develop precancerous lesions. And the second low-potential time point was the four months following exposure, indicating that mice had low stability and high risk of state transition at the time point (i.e. critical state or pre-disease state), which was consistent with the process of LUAD in mice that six months following exposure is the onset of LUAD (Kantrowitz et al. 2018). Combining $V$ and $U$, the groups with similar phenotypic state such as the two months group and the four months group can be distinguished by $U$ score. And the groups with similar homeostatic potential such as the two months group and the six months group can be distinguished by $V$ scores (*p* value = 0.004).

Applying HSM method to the human HCC, mice diabetes, and mice LUAD datasets indicated that it was feasible and effective to use $V$ and $U$ to quantify individual health status. It can distinguish individuals not only for those with similar phenotypic potential by (1) homeostatic potential but also for those with similar homeostatic potential by (2) phenotypic potential.

### Characterizing Homeostatic Potential Contributes to Detect Potential Disease Genes as Well as the Tipping Point or the Pre-Disease State

**Fig. 5:**
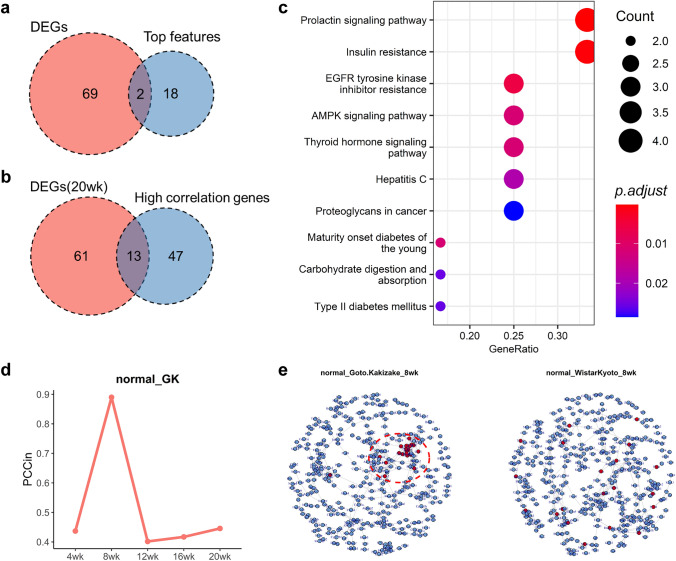
The approach to characterizing homeostatic potential contributed to find potential disease gene. **a** There were only two overlaps between the top 20 genes and the DEGs at eight weeks. **b** There were 13 overlaps between the 74 DEGs at 20 weeks and the 60 genes with high correlation to the top 20 genes. **c** KEGG enrichment analysis of the 13 overlaps showed that they were enriched in multiple pathways associated with diabetes. **d** The average of absolute Pearson correlation coefficients among the top 20 genes (PCCin) at each age, and PCCin was highest at eight weeks. **e** The global correlation network of GK rats and WKY rats at eight weeks, and the correlation among the top 20 genes of GK rats was high

In addition to quantifying individual health status, our approach can also be conducive to detect some potential disease genes based on their contributions to the $U$ score. In the rat diabetes dataset, we have found that the GK rats fed normal diet at eight weeks of age had a low homeostatic potential and high risk of state transition, which is considered as the tipping point or pre-disease state (Chen et al. 2012; Gao et al. 2022; Yang et al. 2018; Zhang et al. 2021). The top 20 genes with the lowest homeostatic potential in the 8-week-old GK rats fed normal diet were identified and considered as important genes in state transition. Then, 71 differential expressed genes (DEGs) were detected between the 8-week-old GK rats fed normal diet and the control WKY rats by Student's *t* test (*p* value < 0.05) (SI. Table 2). There were only two overlapped genes between the top 20 genes with low potential in the 8-week-old GK rats fed normal diet and the 71 DEGs (Fig. 5a). This indicated that the information contained in homeostatic potential and in gene expression or phenotype overlapped in a low degree, which further proved that quantifying individual health status required the combination of the two indexes. And the two overlapped genes may be the key genes in state transition. There were 60 genes to be highly correlated to the top 20 genes ($\left|pcc\right|>0.7$ and $p$-value < 0.01), and there were 14 overlapped genes between the 60 high correlation genes and the 71 DEGs (SI. Table 2). The enrichment analysis was done for these 14 overlapped genes in Kyoto Encyclopedia of Genes and Genomes (KEGG) database (https://www.kegg.jp/). The results showed that they were significantly enriched to "insulin resistance", "AGE-RAGE signaling pathway in diabetic complications" and "Hepatitis C" pathways in KEGG database (SI. Fig. S3), and the three pathways are all related to the occurrence and development of diabetes. Similarly, the DEGs between GK rats and WKY rats at 20 weeks of age were also calculated, because the DEGs at 20 weeks of age were the DEGs between diabetic rats and normal rats. There were 74 DEGs to be identified at 20 weeks of age, and 13 overlapped genes were obtained between the 74 DEGs and the 60 high correlation genes (Fig. 5b and SI. Table 2). The KEGG enrichment analysis of the 13 overlapped genes showed that they were enriched to "Insulin resistance", "AMP-activated protein kinase (AMPK) signaling pathway", "Thyroid hormone signaling pathway" and some other pathways related to diabetes (Fig. 5c). Together, these results suggest that the top 20 genes may be potential disease related genes for T2DM.

In addition, we compared the differences of the high and low homeostatic potential at the network level. The average of absolute Pearson correlation coefficients (PCC) among the top 20 genes $({PCC}_{in})$ was calculated at each timepoint. ${PCC}_{in}$ was calculated by

$${PCC}_{in}=\sum_{i,j}^{n}\left|PCC({x}_{i},{x}_{j})\right|/n, \left(i,j=1,\dots ,n, n=20\right).$$

The ${PCC}_{in}$ at four weeks, 12 weeks, 16 weeks, 20 weeks of age were all between 0.4 and 0.5, while the ${PCC}_{in}$ at 8 weeks of age was as high as 0.89 (Fig. 5d). Meanwhile, the number of edges of the correlation network ($\left|pcc\right|>0.7$ and $p$ value < 0.01) among the top 20 genes was as high as 66 at eight weeks of age, far more than the 0–5 edges at other four timepoints (SI. Fig. S4a). This means that the network between these top 20 genes changed a lot at the age of eight weeks in GK rats, which is also reflected in the comparison of correlation networks at each timepoint we calculated and plotted, which may be one of the reasons for the decrease in homeostatic potential at eight weeks of age (Figs. 5e and SI. S5). We also calculated the dynamic network biomarker (DNB) (Chen et al. 2012; Gao et al. 2022; Yang et al. 2018; Zhang et al. 2021) score of the top 20 genes at five timepoints, ${I}_{DNB}$ in the following equation:

$${I}_{DNB}={SD}_{in}\frac{{PCC}_{in}}{{PCC}_{out}},$$

where ${SD}_{in}$ is the standard deviation of gene expression of the top 20 genes, ${PCC}_{in}$ is the average of absolute PCC among the top 20 genes and ${PCC}_{out}$ is the average of absolute PCC among genes compared within and outside of the top 20 genes (Chen et al. 2012; Gao et al. 2022; Yang et al. 2018; Zhang et al. 2021). The ${I}_{DNB}$ reached a maximum at eight weeks of age, indicating that 8-week age was a critical point for state transition, which was consistent with the calculation of the $U$ scores that characterize homeostatic potential by our approach (SI. Fig. S4c).

## Discussion

In the current work, we proposed an individual health quantification method termed HSM based on the DNB method and diffusion map theory, which could not only distinguish healthy individuals from those within similar phenotypes/states, but also can discriminate different states among health population. The HSM method decomposed the individual health quantification into two parts: characterized phenotypic potential and characterized homeostatic potential. First, the individual health process manifold was constructed based on the phenotype data by LDA-LE. The position on the manifold reflects the current state of each individual, and individuals with close position on the manifold have similar states/phenotypes. In the application of multiple datasets, individuals at different stages had markedly different positions/states on the health state manifold. We also compared LDA-LE with Linear discriminant analysis (LDA), Principal components analysis (PCA) and Multidimensional scaling (MDS) in the supplementary material. The results suggested that the LDA-LE is similar with PCA and MDS, and slightly better than LDA for quantifying individual health. Second, the homeostatic potential of the individual was characterized by the minimum principal curvature of the homeostatic potential landscape constructed by the strongly correlated subnet, which could measure the stability of the individual in the current state based on DNB theory. Individuals with low homeostatic potential had low stability and high risk to transfer into the adjacent stage on the whole health process manifold. In the case of Roux-en-Y Gastric Bypass surgery, individuals with low homeostatic potential had higher BMI or plasma glucose. In the other cases, the stages with low homeostatic potential were the tipping points or critical state just before the disease occurred. And we validated the accuracy of $U$ score on simulated data in the supplementary material. The accuracy was 0.85 when the sample size was three, and increased with the sample size.

The method can not only accomplish group-wise comparisons of the health state, but more importantly, also can quantify and track individual health status. Since the distance of an individual between the current state and the disease state, and the stability of the current state can be quantified, it is possible to determine improvement or deterioration of individual health status over time or with treatment, which facilitates the targeted prevention and intervention of diseases. In addition, our approach also contributes to find the potential disease related genes of various diseases.

There are still some features and issues to be discussed: (1) our body is a complicated system formed by each tissue, pathway and organ performing its own functions and complementing each other. Since most diseases affect only certain tissues and pathways, not all molecular expressions in a disease state are different from that in a healthy state. Therefore, features related to target diseases should be screened out first to reduce noise and disturbance before quantifying individual health status with this approach, especially before quantifying homeostatic potential. And the data are preferably measured from tissue related to the target disease because of tissue specificity. (2) In our approach, the quantification of individual homeostatic potential is based on multi-samples data at a same state. The $U$ score that characterizes homeostatic potential of the individual without multi-samples data cannot be calculated separately, but be calculated based on all individuals of the stage or group. Further improvements of our approach on the quantification for the homeostatic potential of the individual without multi-samples data will be performed as our future work.

In summary, by DNB method and diffusion map theory, we provided a new approach to quantify and evaluate individual health status, which well combines phenotypic and homeostatic features. To reveal the essential state transition laws of individual health status needs much more efforts through joint experimental and theoretical studies in the future.

## Conclusion

Here, we have developed a new method termed HSM for quantifying individual health state, and it can be used to evaluate individual health state in two features. By applying multiple public datasets, we demonstrated that HSM method was able to quantify the health states or distinguish health differences not only for the individuals with a similar phenotype by homeostatic potential, but also for the individuals with a similar homeostatic potential by phenotypic potential. The HSM method can describe the individual health state during not only disease development process but also recovery process, which has the potential to contribute to early signals warning of diseases and prevention or intervention in the early stage of diseases.

## Supplementary Information

Below is the link to the electronic supplementary material.

Supplementary file1 (DOCX 21 KB)

Supplementary file2 (PDF 18792 KB)

Supplementary file3 (XLSX 32 KB)

Supplementary file4 (XLSX 12 KB)

Supplementary file5 (XLSX 10 KB)

Supplementary file6 (XLSX 10 KB)

Supplementary file7 (XLSX 10 KB)

Supplementary file8 (XLSX 10 KB)

Supplementary file9 (XLSX 10 KB)

## Data Availability

The Rats diabetes dataset, the human HCC dataset, the mice diabetes dataset and the mice LUAD dataset are available at NCBI's GEO database under the accession number: GSE13270, GSE6764, GSE15150, GSE102707. The human Roux-en-Y Gastric Bypass dataset is available at PRIDE partner repository (identifier PXD008071).
