# Supplementary material for: Quantifying Individual Health Status from Multi-omics Data by Health State Manifold
Source: Phenomics. 2025 Dec 15;5(5):469–86. doi: 10.1007/s43657-024-00188-4 (PMC12881232; doi:10.1007/s43657-024-00188-4)
Supplement: Supplementary file 1 — Supplementary file1 (DOCX 21 KB) [file 43657_2024_188_MOESM1_ESM.docx]

**Derivation.** The derivation of the target function of Laplacian Eigenmaps

The target function of Laplacian Eigenmaps is as formula:

$$\begin{aligned} \min_{} \sum_{i,j} \left\| y_{i}-y_{j} \right\|^{2}W_{ij}, \left( i,j=1,\ldots,n+m \right)\# \end{aligned}$$

And

$$\begin{aligned} \min_{} \sum_{i,j} \left\| y_{i}-y_{j} \right\|^{2}W_{ij}=\sum_{i} \sum_{j} \left( y_{i}^{T}y_{i}-2y_{i}^{T}y_{j}+y_{j}^{T}y_{j} \right)W_{ij} \\ =\sum_{i} \left( \sum_{j} W_{ij} \right)y_{i}^{T}y_{i}+\sum_{j} \left( \sum_{i} W_{ij} \right)y_{j}^{T}y_{j}-2\sum_{i} \sum_{j} y_{i}^{T}y_{j}W_{ij} \\ =2\sum_{i} D_{ii}y_{i}^{T}y_{i}-2\sum_{i} \sum_{j} y_{i}^{T}y_{j}W_{ij} \\ =2\sum_{i} \left( \sqrt{D_{ii}}y_{i} \right)^{T}\left( \sqrt{D_{ii}}y_{i} \right)-2\sum_{i} y_{i}^{T}\left( \sum_{j} y_{j}W_{ij} \right) \\ =2trace\left( Y^{T}DY \right)-2\sum_{i} y_{i}^{T}\left( YW \right)_{i} \\ =2trace\left( Y^{T}DY \right)-2trace\left( Y^{T}WY \right) \\ =2trace\left[ Y^{T}\left( D-W \right)Y \right]=2trace{(Y}^{T}LY) \end{aligned}$$

where $W$ is the adjacency matrix of $X$ constructed by person correlation coefficients (PCC), $L$ is the Laplacian matrix, $L=D-W$, $(D_{ii}=\sum_{j=1}^{n+m} W_{ij})$, and diagonal matrix $D$ is the degree matrix.

**Simulation**. We applied the approach on simulated dataset to evaluate the accuracy of U scores. The individual molecular expression was simulated by the bivariate normal distribution and we generated $n$ random samples from the bivariate normal distributions $N(0,0,1,1,0)$ and $N(0,0,3,3,0)$, respectively. The smaller variance of the bivariate normal distribution in the simulated molecular expression characterized the higher homeostatic resilience. The molecular expression can also be simulated by a higher dimensional normal distribution, and here we used a two-dimensional normal distribution for the convenience of calculation. Then we calculated two $U$ scores based on both sets of random samples. If the $U$ score for $N(0,0,1,1,0)$ is greater than the $U$ score for $N(0,0,3,3,0)$, the result of this simulation is positive, and vice versa. The simulation was repeated 10,000 times, and the accuracy with $n=3,4,\ldots,10$ is given in SI. Fig. S6. The results show that the accuracy increases with sample size $n$. When $n$= 3, the accuracy is 0.850, accuracy 0.930 for $n$= 5, and 0.985 for $n$= 10.

**The advantage of** $\boldsymbol{U}$ **score compared with DNB** **score.** The function or explanation of the $U$ score is similar to the DNB score, especially in the terms of detecting critical point for complex diseases, and actually the $U$ score can be considered as one of DNB scores due to the similar criteria or principles (e.g. bifurcation) applied in both scores. But their quantification measurement are different. The rat T2D example shows that both $U$ score and DNB score provided similar strong signals at 8 weeks and 20 weeks which are the two critical points during rat T2D progression (Fig. 2c and SI. Fig. S7a and SI. Fig. S7b). The DNB score is commonly used for providing early warning signals on complex diseases, while the $U$ score is more sensitive in measuring individual homeostatic resilience. And $U$ score performs slightly better than the DNB score in the recovery ability from disease to health. For example, we applied both $U$ and DNB scores on the dataset GSE88794, which contains 39 healthy, overweight men and women who were subjected to a mixed meal test (MMT) before and after a 12 weeks positive intervention with a 20% energy restriction (ER) diet. Total RNA from peripheral blood mononuclear cells (PBMCs) was isolated at fasting, and at postprandially during the MMT at 60, 120, 240 and 360 min. And the body fat was measured before and after the intervention. We calculated the $U$ score and DNB score separately for each individual with the RNA data before intervention. The difference of body fat before and after the intervention (∆body fat) was calculated for each individual. Then, we divided the population into two groups according to the mean of $U$ scores of all individuals and compared the ∆body fat between the two groups. The *t*-test result showed that the body fat of the group with lower $U$ scores decreased significantly even more ($pvalue=0.058$), suggesting that individuals with lower homeostatic potential responded more strongly to the positive intervention and the effect of the intervention was better (SI. Fig. S7c). But the ∆body fat was not significantly different between groups with different DNB scores ($pvalue=0.173$) (SI. Fig. S7d). These results indicate that although $U$ score and DNB score are similar in detecting critical point of a complex disease, $U$ score performs better in the recovery process from disease to health, and $U$ score may have the potential to predict the effect of intervention, which is very important for the treatment and early intervention of diseases.

**The relation between** $\boldsymbol{U}$**,** $\boldsymbol{V}$ **derived from different tissue context.** In fact, there are differences between the $U$, $V$ derived from the context of different tissues because of tissue specificity. For example, we applied the HSM method on datasets GSE13270 and GSE13269, which are from different tissues in the same study for rats T2D. The dataset GSE13270 is from rat liver tissue and GSE13269 is from rat gastrocnemius muscle tissue. The Pearson correlation coefficient of the $V$ scores derived from the two tissues is 0.92 (SI. Fig. S9a-b), which means that the $V$ scores are similar for the data from different tissues. But the $U$ scores from the two tissues are different. In liver tissue, the strong signals of $U$ scores first appeared at the age of 8 weeks and then at 20 weeks (SI. Fig. S9c), while in muscle tissue, the strong signals of $U$ scores first appeared at the age of 12 weeks and then at 20 weeks (SI. Fig. S9d). The results mean that the $U$ is more tissue-specific than $V$, probably because the $U$ score describes the local state. However, the strong signal at 20 weeks was detected by $U$ scores of both tissues in despite of the tissue specificity, which suggests that it is better to use the tissue closely related to the disease in clinical application. But it is also effective to use tissue that is easier for sampling, such as blood.

**Compare the LDA-LE, LDA, PCA, MDS****.** The LDA-LE method was derived from the LDA combined with the Laplacian eigenmaps. It not only separates the different groups, but also makes the samples with close distances still have close distances after dimension reduction. Therefore, the LDA-LE is suitable for quantifying individual health. We compared LDA-LE with LDA, PCA and MDS by rats T2DM dataset, and used the Adjust-$R^{2}$ as the criterion. We ranked the groups in rats T2DM dataset from 1 to 20 in order of disease development and chronological order. Samples from the same group had the same sequence number. For example, the group of GK rats at 4 weeks with normal diet was NO.1, and the sequence number of all samples in this group was 1. Then we calculated the Adjust-$R^{2}$ between the ranking and the data after dimension reduction. The Adjust-$R^{2}$ from LDA-LE is 0.7176, the Adjust-$R^{2}$ from LDA is 0.5173, the Adjust-$R^{2}$ from PCA is 0.6744 and the Adjust-$R^{2}$ from MDS is 0.67 (SI. Fig. S10). The results suggested that the LDA-LE is similar to PCA and MDS, and slightly better than LDA for quantifying individual health. And We have added this section to supplemental materials in the revised manuscript.

In addition, the LDA-LE is part of our HSM method but not all of it. The main novelty of HSM is to quantify individual health with two key features, (1) homeostatic potential estimated by curvature and (2) phenotypic potential estimated by LDA-LE.
