# Supplementary material for: Quantifying Individual Health Status from Multi-omics Data by Health State Manifold
Source: Phenomics. 2025 Dec 15;5(5):469–86. doi: 10.1007/s43657-024-00188-4 (PMC12881232; doi:10.1007/s43657-024-00188-4)
Supplement: Supplementary file 2 — Supplementary file2 (PDF 18792 KB) [file 43657_2024_188_MOESM2_ESM.pdf]

SI. Fig. S1

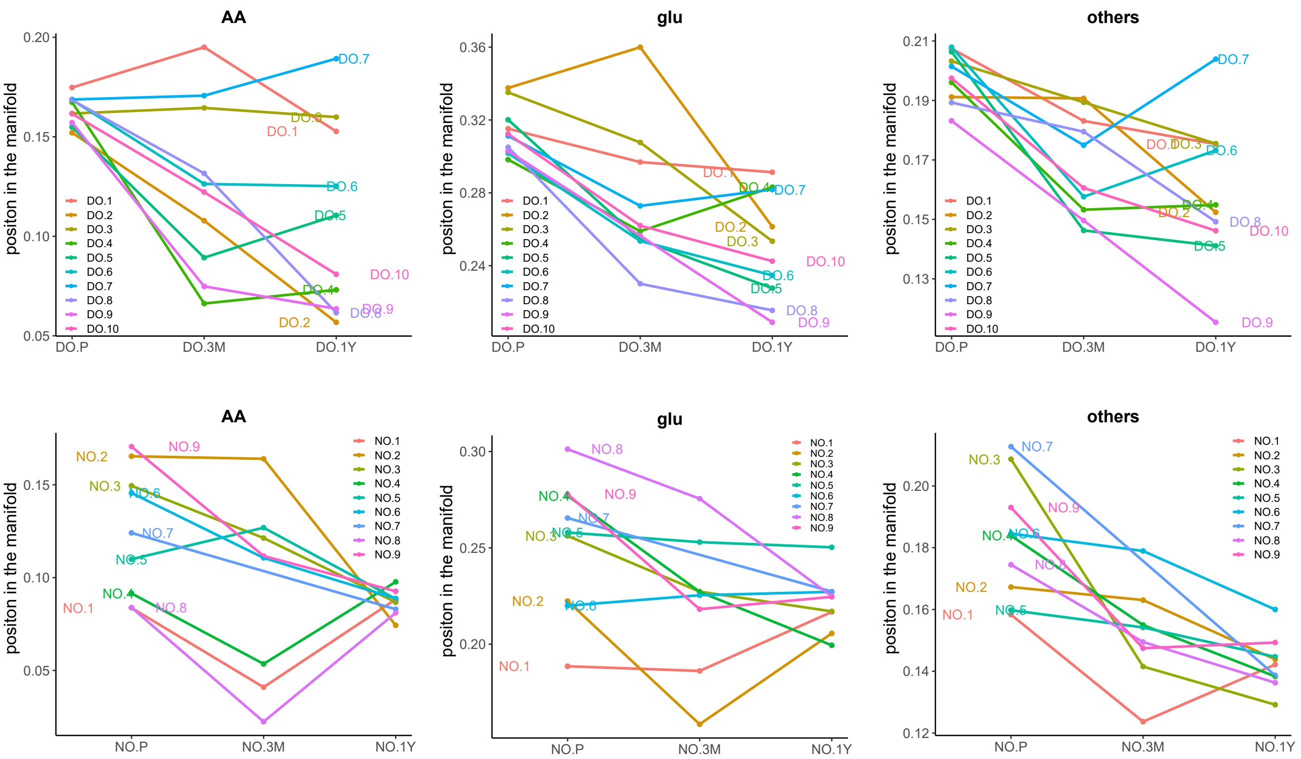

SI. Fig. S1 Each individual's position (–V score) in the each axis of health state manifold at the before, three-months and one-year after RYGB stages decreased gradually with recovery time

SI. Fig. S2

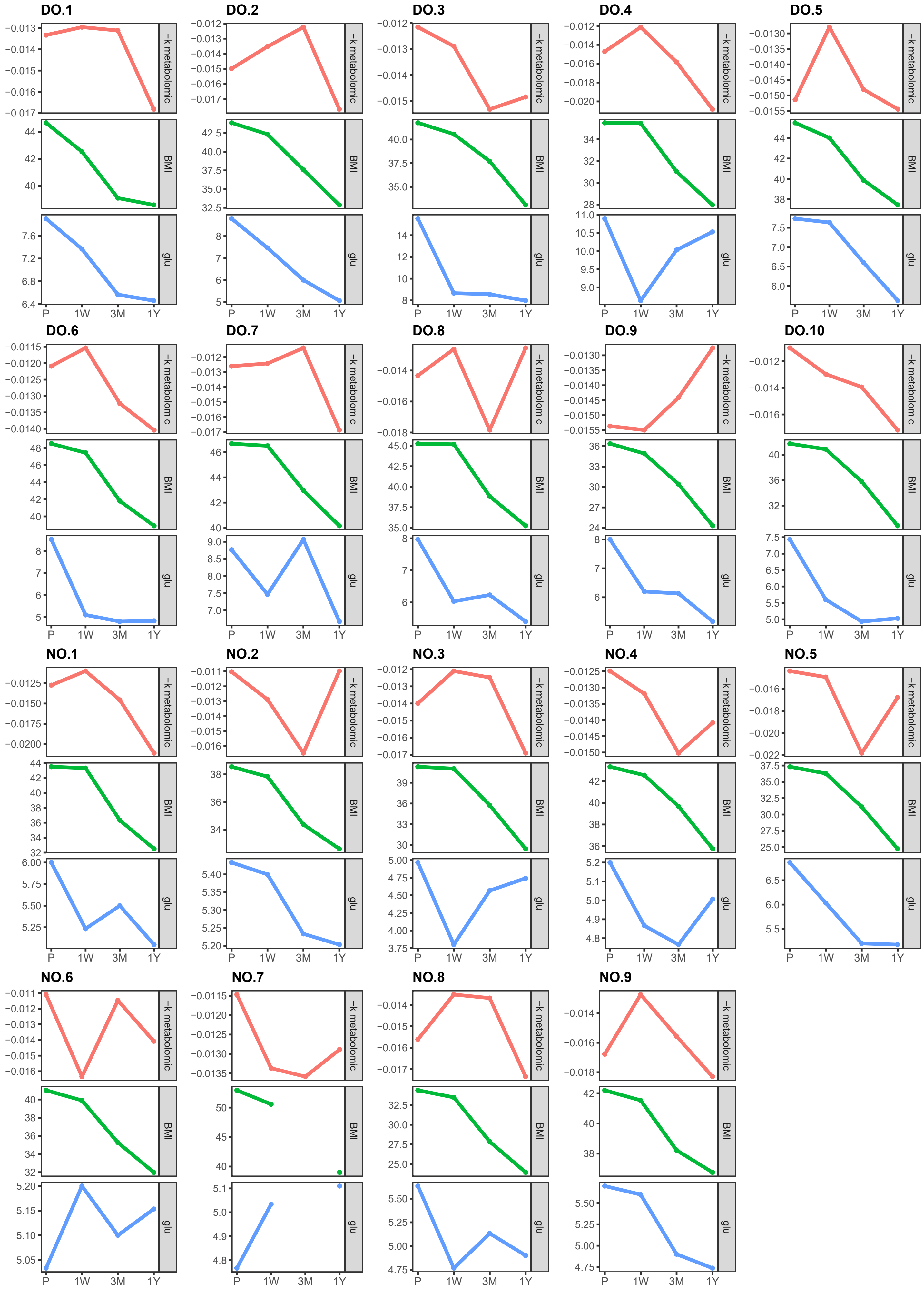

SI. Fig. S2 The –U score of each individual changed with postoperative recovery time in line with the changes of their BMI and plasma glucose

SI. Fig. S3

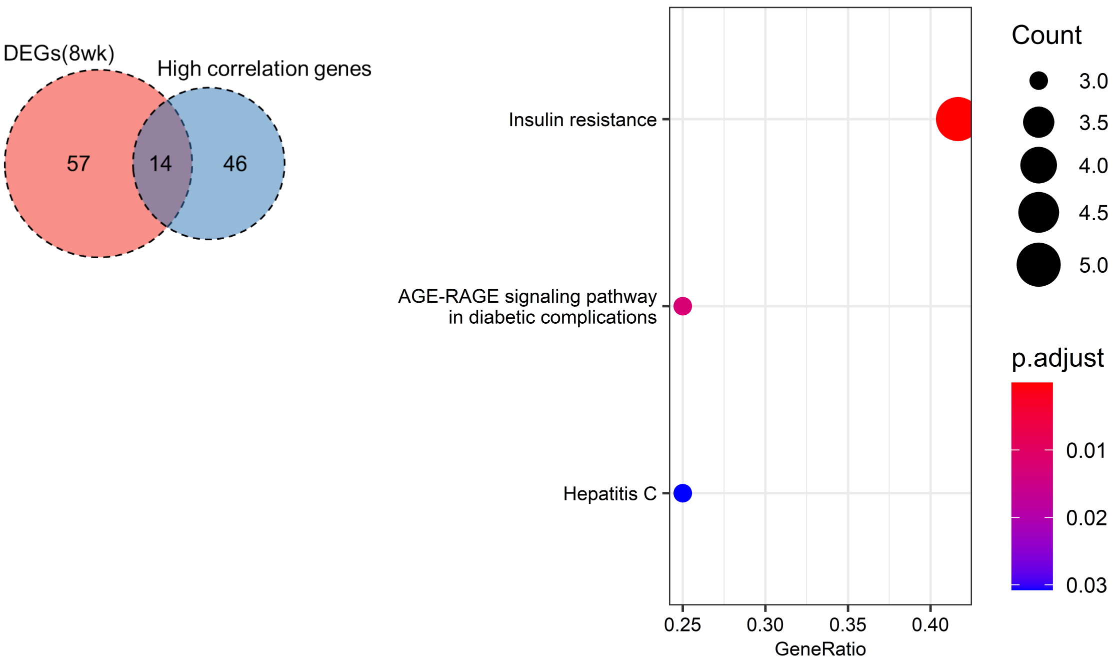

SI. Fig. S3 There were 14 overlaps between the 60 genes with high correlation and the 71 DEGs. KEGG enrichment analysis of these 14 genes showed that they were enriched in "insulin resistance", "AGE-RAGE signaling pathway in diabetic complications" and "Hepatitis C"

SI. Fig. S4

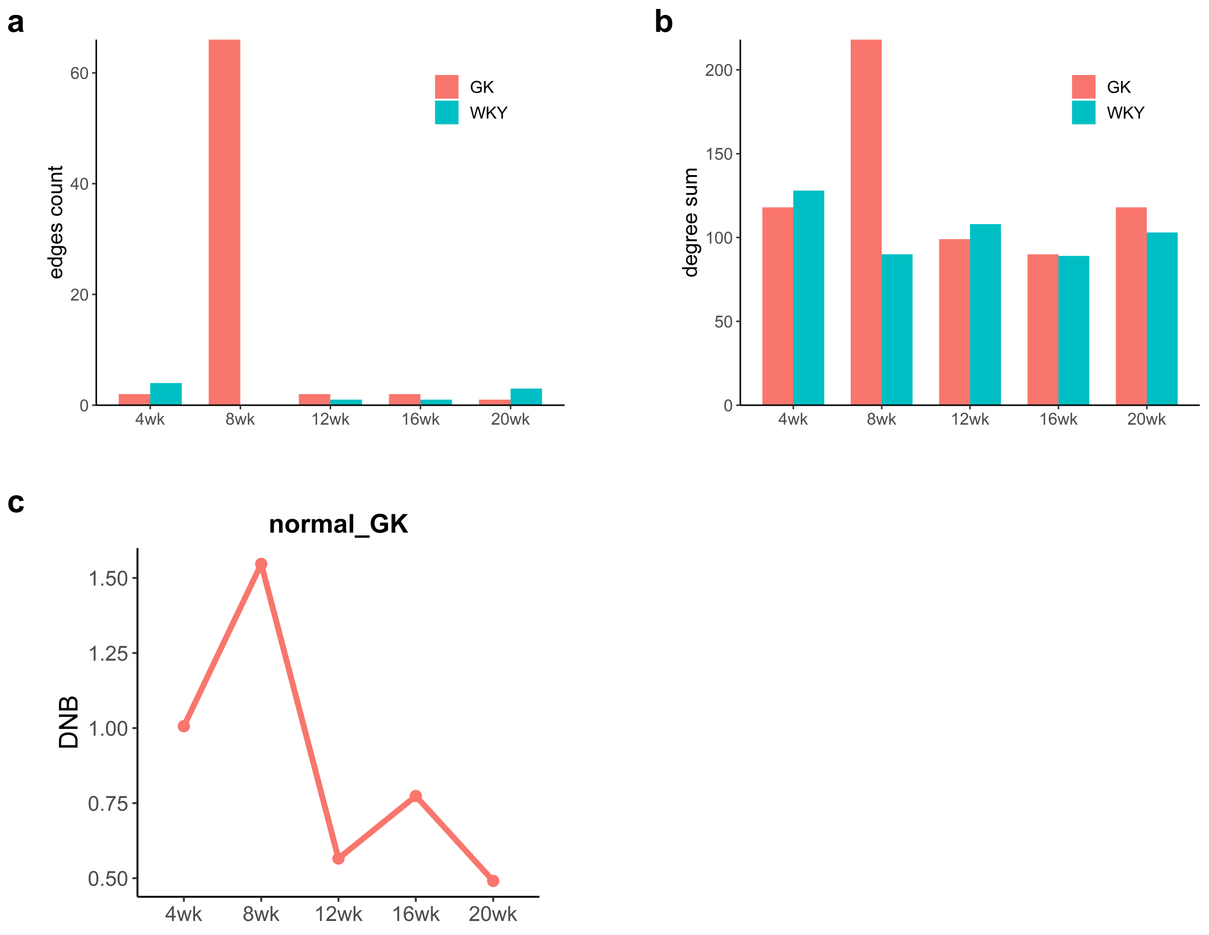

SI. Fig. S4 **a** the number of edges of the correlation network (|pcc|>0.7 and p-value<0.01) between the top20 genes was as high as 66 at 8 weeks of age, far more than the 0 to 5 edges at other four timepoints. **b** The sum of the degrees of the top20 genes (8 weeks) in the global correlation network at each age. The difference between GK rats and WKY rats was greatest at 8 weeks. **c** DNB scores of GK rats at each age, and reached a maximum at 8 weeks of age

SI. Fig. S5

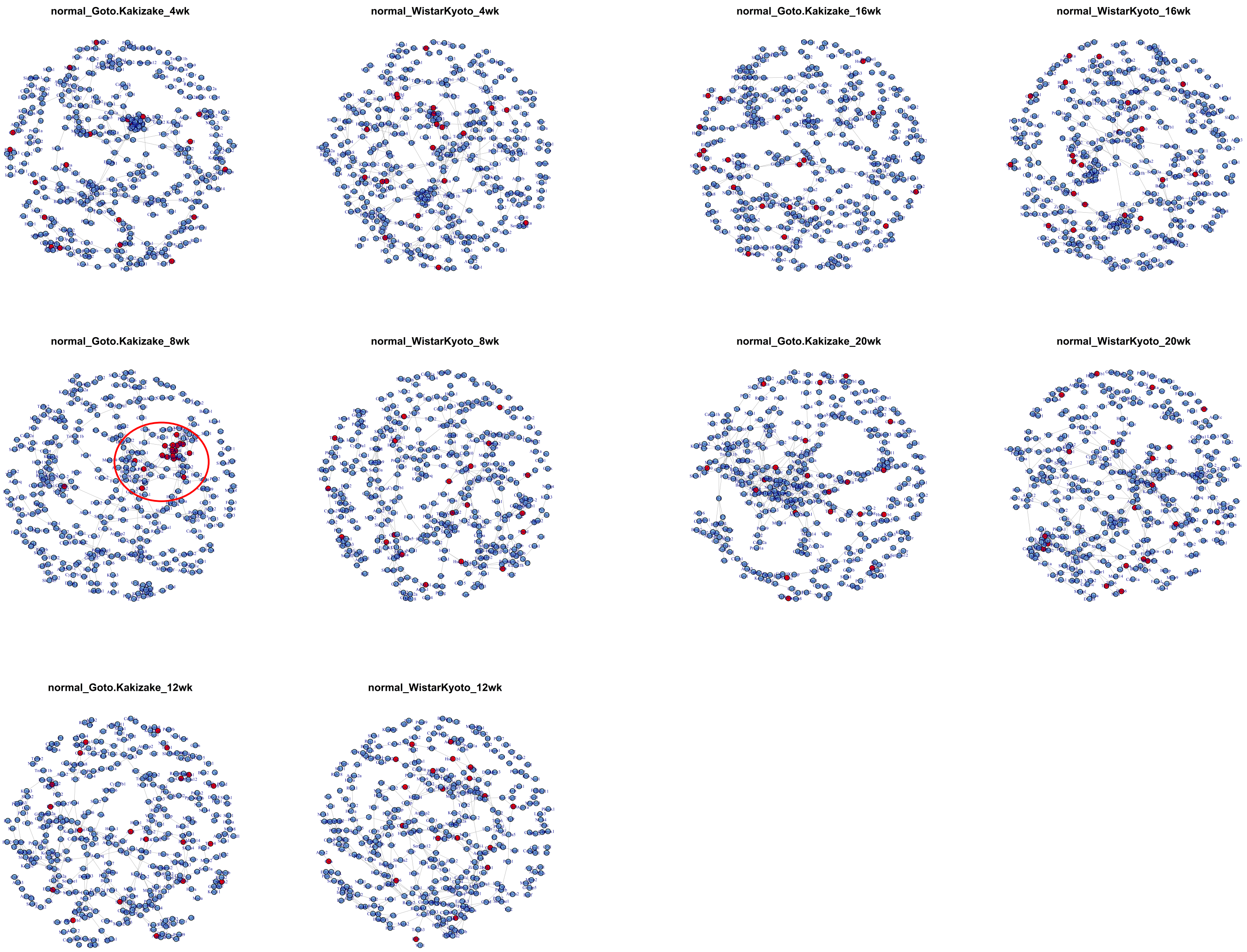

SI. Fig. S5 Global correlation networks of 362 genes of GK rats and WKY rats at each age. The red dots were the top20 genes for 8 weeks. Only in the network of 8-week-old GK rats, these top20 genes were highly correlated to each other

SI. Fig. S6

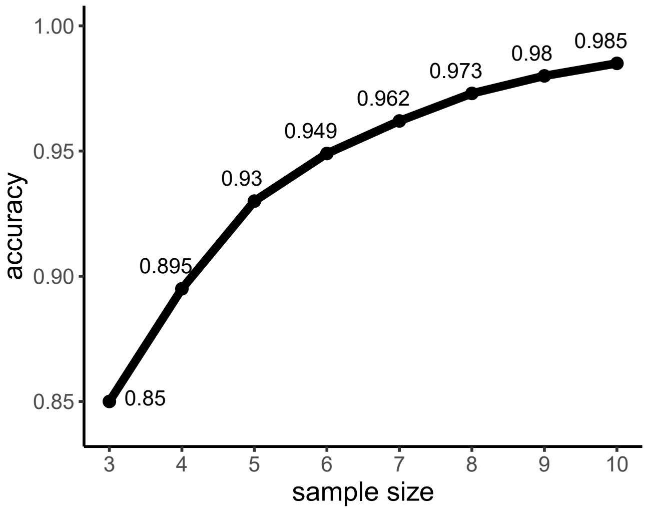

SI. Fig. S6 The accuracy of U scores with different sample sizes from the simulated data. The accuracy increases with sample size

SI. Fig. S7

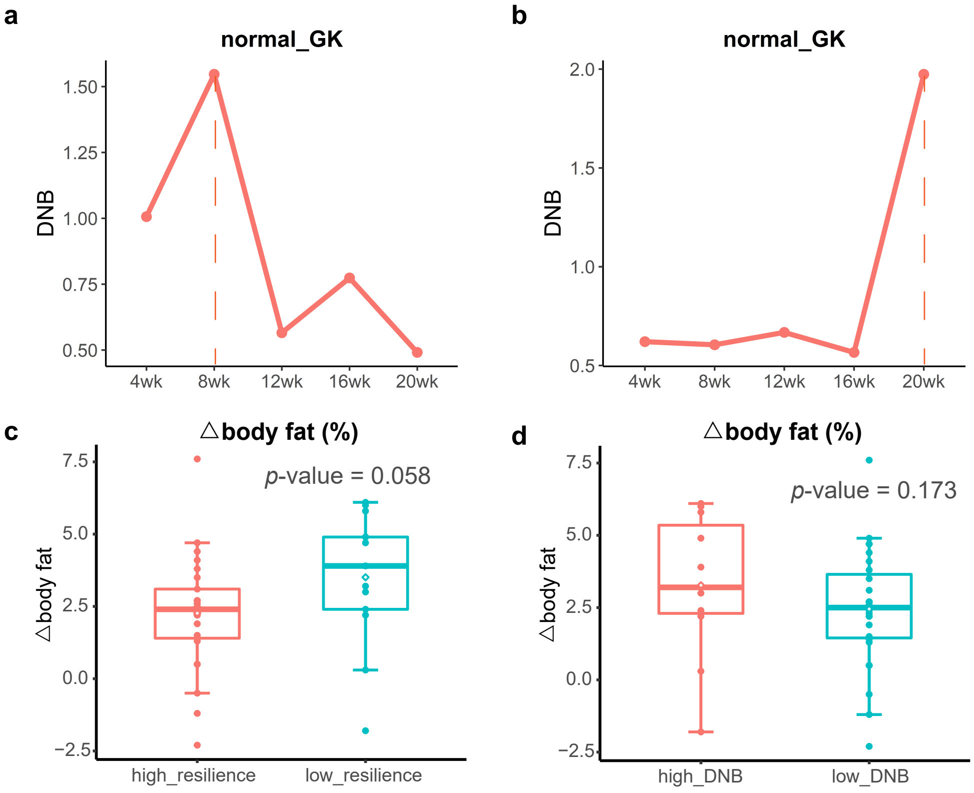

SI. Fig. S7 Comparing U score with DNB score. **a** The strong signal of the DNB for L1 gene module from GK rats with normal diet appeared at 8 weeks, consistent with the signal of U scores. **b** The strong signal of the DNB score for L2 gene module from GK rats with normal diet appeared at 20 weeks, consistent with the signal of U scores. **c** Individuals with low-resilience had a significantly greater reduction in body fat after the intervention than those with high-resilience (*p*-value = 0.058, *t*-test). **d** There was no significant difference in the reduction of body fat between individuals with high-DNB scores and individuals with low-DNB scores

SI. Fig. S8

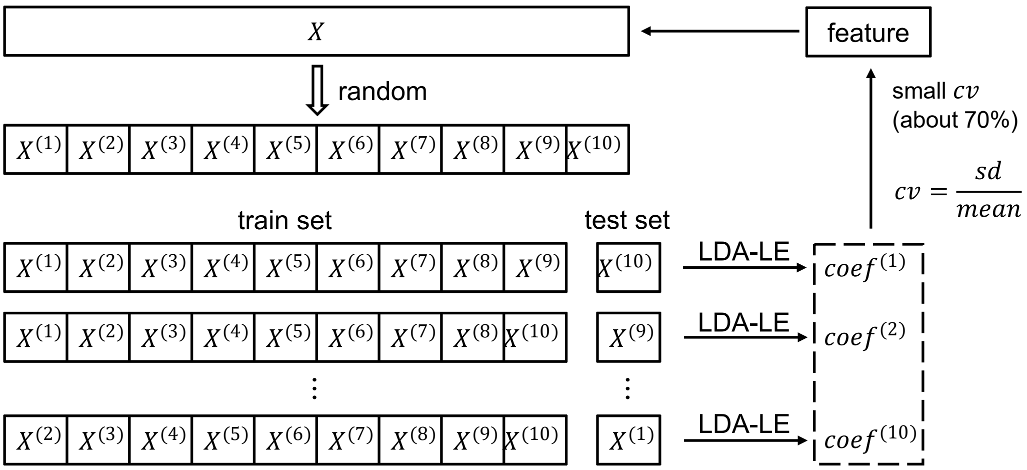

SI. Fig. S8 The flowchart of tenfold cross-validation. Dividing ***X*** randomly into ten parts, take nine parts as the training set to train the model by LDA-LE and another as the test set. Obtain the regression coefficient of each feature in this model. Repeat ten times so that ten coefficients of each feature were obtained. Compute the ***cv*** of the ten coefficients for each feature. The features with small ***cv*** (about the 70 percent) are considered as stable features and are screened out for training the model of health process manifold

SI. Fig. S9

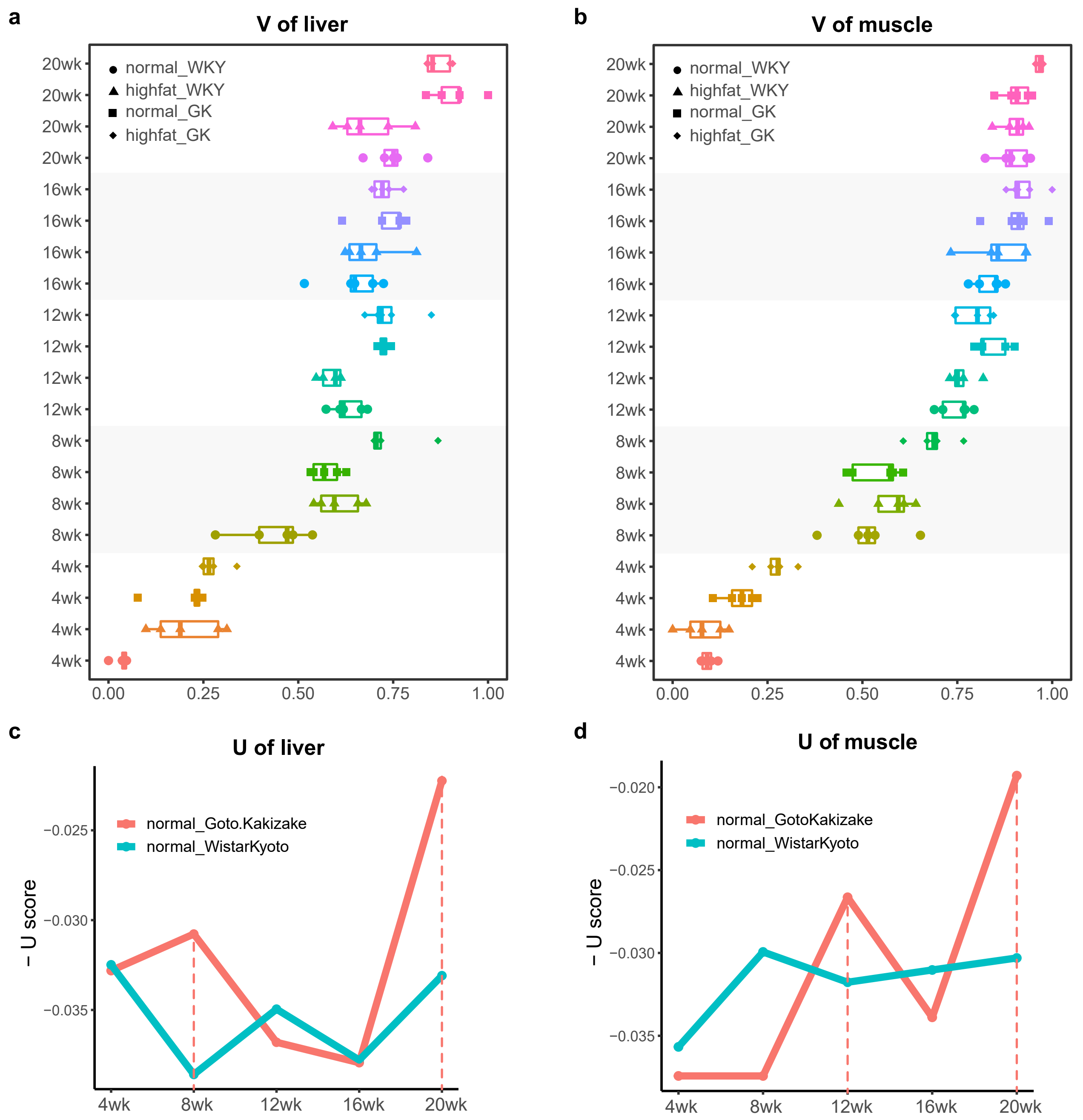

SI. Fig. S9 The U and V derived from the context of two different tissues. There are differences between the U, V derived from different tissues because of tissue specificity. **a, b** The V scores derived from two tissues were similar and the Pearson correlation coefficient is 0.92. **c, d** The U scores derived from two tissues. The first strong signals of U scores for two tissues appeared at different stages, suggesting that U is more tissue-specific than V for such cases

SI. Fig. S10

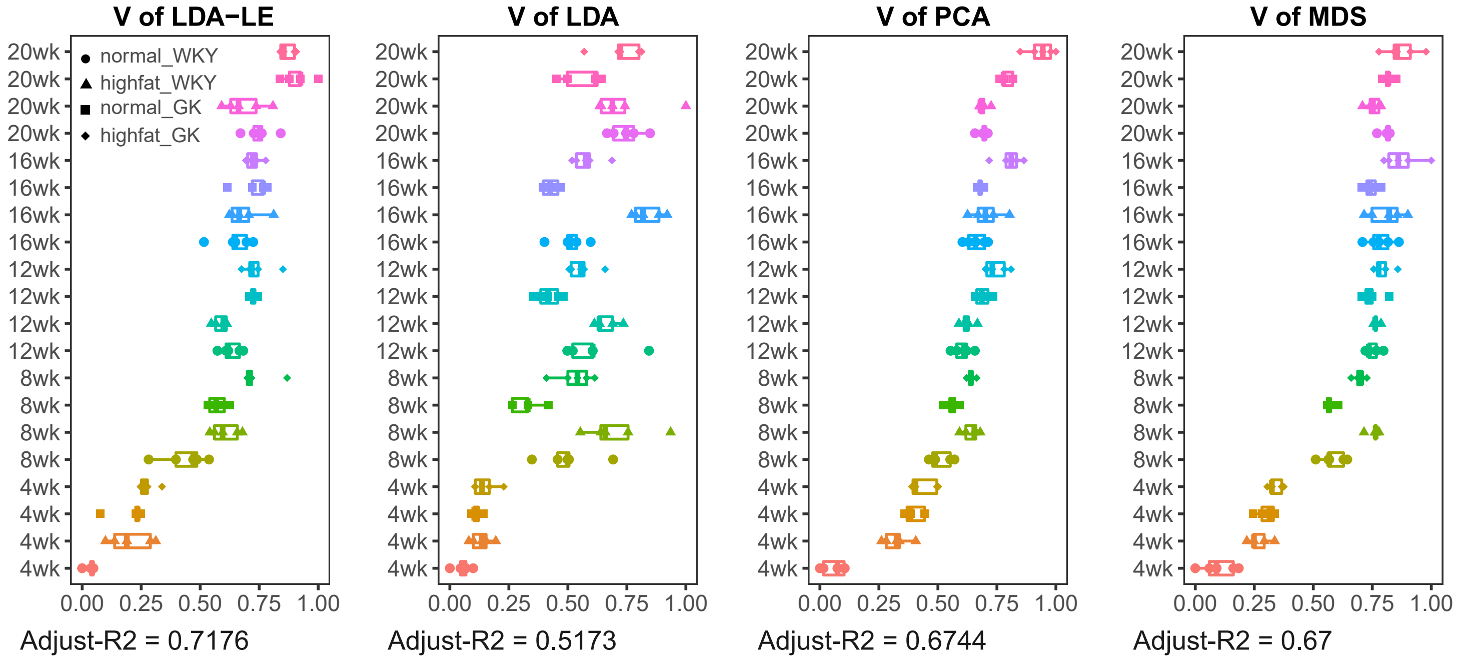

SI. Fig. S10 Comparing LDA-LE with LDA, PCA and MDS for quantifying individual health. The Adjust-R2 between the sequence number ranked in order of disease development and the V scores of different methods were calculated. The Adjust-R2 from LDA-LE is 0.7176, the Adjust-R2 from LDA is 0.5173, the Adjust-R2 from PCA is 0.6744 and the Adjust-R2 from MDS is 0.67. The results suggested that the LDA-LE is similar to PCA and MDS, and slightly better than LDA for quantifying individual health
